# Assessing professional identity formation (PIF) amongst medical students in Oncology and Palliative Medicine postings: a SEBA guided scoping review

**DOI:** 10.1186/s12904-022-01090-4

**Published:** 2022-11-18

**Authors:** Kelly Jia Hui Teo, Mac Yu Kai Teo, Anushka Pisupati, Rui Song Ryan Ong, Chloe Keyi Goh, Claire Hui Xian Seah, You Ru Toh, Neha Burla, Natalie Song Yi Koh, Kuang Teck Tay, Yun Ting Ong, Min Chiam, Warren Fong, Limin Wijaya, Suzanne Pei Lin Goh, Lalit Kumar Radha Krishna

**Affiliations:** 1 Yong Loo Lin School of Medicine, National University of Singapore, 1E Kent Ridge Road, NUHS Tower Block, Level 11, Singapore, 119228 Singapore; 2 Division of Supportive and Palliative Care, National Cancer Centre Singapore, 11 Hospital Crescent, Singapore, 16961 Singapore; 3 Division of Cancer Education, National Cancer Centre Singapore, 11 Hospital Crescent, Singapore, 169610 Singapore; 4 Duke-NUS Medical School, 8 College Road, Singapore, 169857 Singapore; 5 Department of Rheumatology and Immunology, Singapore General Hospital, 16 College Road, Block 6 Level 9, Singapore General Hospital, Singapore, 169854 Singapore; 6 Division of Infectious Disease, Singapore General Hospital, 16 College Road, Block 6 Level 7, Singapore General Hospital, Singapore, 169854 Singapore; 7 KK Women’s and Children Hospital, 100 Bukit Timah Rd, Singapore, 229899 Singapore; 8 Palliative Care Institute Liverpool, Academic Palliative & End of Life Care Centre, Cancer Research Centre, University of Liverpool, 200 London Road, Liverpool, L3 9TA UK; 9 Centre for Biomedical Ethics, National University of Singapore, Blk MD11, 10 Medical Drive, #02-03, Singapore, 117597 Singapore; 10 The Palliative Care Centre for Excellence in Research and Education, PalC C/O Dover Park Hospice, 10 Jalan Tan Tock Seng, PalC, 308436 Singapore; 11 Health Data Science, University of Liverpool, Whelan Building, The Quadrangle, Brownlow Hill, Liverpool, L69 3GB UK

**Keywords:** Professional identity formation, Medical students, Palliative care, Oncology, Personhood, Medical school, Identity, Mentoring

## Abstract

**Background:**

Introduction to a multi-professional team who are working and caring for the dying, and facing complex moral and ethical dilemmas during Oncology and Palliative Medicine postings influence a medical student’s professional identity formation (PIF). However, limited appreciation of PIF, inadequate assessments and insufficient support jeopardise this opportunity to shape how medical students think, feel and act as future physicians. To address this gap, a systematic scoping review (SSR) of PIF assessment methods is proposed.

**Methods:**

A Systematic Evidence-based Approach (SEBA) guided SSR of assessments of PIF in medical schools published between 1^st^ January 2000 and 31^st^ December 2021 in PubMed, Embase, ERIC and Scopus databases was carried out. Included articles were concurrently content and thematically analysed using SEBA’s Split Approach and the themes and categories identified were combined using SEBA’s Jigsaw Perspective. The review hinged on the following questions: *“what is known about the assessment of professional identity formation amongst medical students?”, “what are the theories and principles guiding the assessment of professional identity formation amongst medical students?”, “what factors influence PIF in medical students?”, “what are the tools used to assess PIF in medical students?”, and “what considerations impact the implementation of PIF assessment tools amongst medical students?”*.

**Results:**

Two thousand four hundred thirty six abstracts were reviewed, 602 full-text articles were evaluated, and 88 articles were included. The 3 domains identified were 1) theories, 2) assessment, and 3) implementation in assessing PIF. Differing attention to the different aspects of the PIF process impairs evaluations, jeopardise timely and appropriate support of medical students and hinder effective implementation of PIF assessments.

**Conclusion:**

The Krishna-Pisupati model combines current theories and concepts of PIF to provide a more holistic perspective of the PIF process. Under the aegis of this model, Palliative Care and Oncology postings are envisaged as Communities of Practice influencing self-concepts of personhood and identity and shaping how medical students see their roles and responsibilities as future physicians. These insights allow the forwarding of nine recommendations to improve assessments of PIF and shape the design of a PIF-specific tool that can direct timely and personalized support of medical students.

**Supplementary Information:**

The online version contains supplementary material available at 10.1186/s12904-022-01090-4.

## Introduction

Oncology and Palliative Medicine postings help medical students understand [1–3], learn, practice and develop their ethics [4], empathy [5], communication [6], professionalism [7], legal [8, 9] and collaborative skills and competencies [10]. These postings also offer an opportunity for medical students to witness, discuss and participate in complex clinical decision-making [11] on matters such as end-of-life goals, best interests determinations, quality-of-life, and desired places of care and death [12]. With data suggesting that such experiences impact how medical students conceive their roles, responsibilities, competencies, and conduct within the team and the manner that they see, feel and act as future physicians, the role of Oncology and Palliative Medicine postings in influencing the professional identity formation (PIF) of medical students has come under the spotlight [13–15].

With potential impact on future practice, professionalism, teamworking and service orientation, the importance of shaping PIF is clear [16, 17]. Thus, the evidence that experiences in Oncology and Palliative Medicine postings influence a medical student’s PIF offers just such an opportunity to mould PIF [13–15]. This calls for better assessments of PIF in Oncology and Palliative Medicine postings. To be clear rather than being concerned with remediation and maintaining competencies, proposed assessments of PIF during Oncology and Palliative Medicine postings aim to direct support to medical students in a timely, personalised and appropriate manner.

## Methodology

**Fig. 1:**
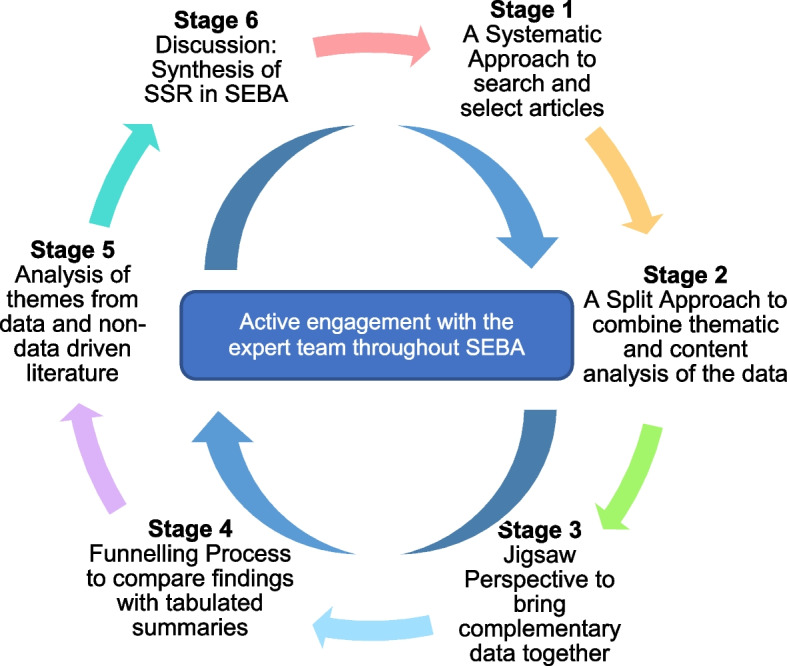
The SSR in SEBA Process

In the absence of a consistent approach to assess PIF longitudinally and holistically [16, 17], a Systematic Evidenced Based Approach guided systematic scoping review (henceforth SSR in SEBA) is proposed to map current assessments of PIF amongst medical students. This SSR in SEBA is overseen by an expert team comprised of medical librarians from the Yong Loo Lin School of Medicine (YLLSoM) and the National Cancer Centre Singapore (NCCS), and local educational experts and clinicians at NCCS, the Palliative Care Institute Liverpool, YLLSoM and Duke-NUS Medical School who guide, oversee and support all stages of SEBA in order to ensure the transparency, accountability and reproducibility of this review [5, 18–28]. This SSR in SEBA is also shaped by SEBA’s constructivist ontological perspective and relativist lens as well as the principles of interpretivist analysis to enhance reflexivity of the analysis and the discussions of this SSR in SEBA [29–32] Fig. 1.

### Stage 1 of SEBA: systematic approach

#### Determining the title and research question and inclusion criteria

The PICOs format was employed (Table 1) to guide the primary research question which is “What is known about the assessment of professional identity formation amongst medical students?” and the secondary research questions are “What are the theories and principles guiding the assessment of professional identity formation amongst medical students?”, “What factors influence PIF in medical students?”, “What are the tools used to assess PIF in medical students?”, and “What considerations impact the implementation of PIF assessment tools amongst medical students?”.

**Table 1:** PICOs, Inclusion Criteria and Exclusion Criteria Applied to Database Search

PICOs	Inclusion Criteria	Exclusion Criteria
Population	• Undergraduate medical schools • Postgraduate medical schools	• All qualified physicians (including residents) within the postgraduate clinical, medical, research and/ or academic settings without the mention of medical schools • Allied health specialties such as Pharmacy, Dietetics, Chiropractic, Midwifery, Podiatry, Speech Therapy, Occupational and Physiotherapy without the mention of medical schools • Non-medical specialties such as Clinical and Translational Science, Alternative and Traditional Medicine, Veterinary, Dentistry without the mention of medical schools
Intervention	• Methods of assessing professional identity formation of medical students	NA
Comparison	• Comparisons of the assessment tools, including the assessment principles, modalities and criteria	NA
Outcome	• Assessment principles, modalities and criteria • Impact of assessment on those being assessed	NA
Study design	• Articles in English or translated to English • Grey Literature / electronic and print information not controlled by commercial publishing • All study designs including: ◦ Mixed methods research, meta-analyses, systematic reviews, randomized controlled trials, cohort studies, case–control studies, cross-sectional studies, and descriptive papers • Year of Publication: 1st Jan 2000 – 31st Dec 2021 • Databases: PubMed, Embase, ERIC, Scopus	• Case reports and series, ideas, editorials, conference abstracts, and perspectives

#### Searching

Independent searches of PubMed, Embase, ERIC and Scopus were conducted between 13^th^ February 2022 and 18^th^ April 2022. To ensure a viable and sustainable research process, the research team confined the searches to articles published between 1^st^ January 2000 to 31^st^ December 2021 to account for prevailing manpower and time constraints. Additional ‘snowballing’ of references of the included articles ensured a comprehensive review of articles in the field [33]. The full search strategy can be found in Appendix A.

#### Extracting and charting

Using an abstract screening tool, the research team independently reviewed abstracts to be included and employed ‘negotiated consensual validation’ to achieve consensus on the final list of articles to be included [34].

### Stage 2 of SEBA: split approach

The Split Approach [35] employs concurrent thematic and directed content analysis of the included full-text articles as well as the creation of tabulated summaries of these articles. This process is carried out by three independent teams. The first team summarised and tabulated the included full-text articles according to recommendations set out by Wong, Greenhalgh [36]’s RAMESES publication standards and Popay, Roberts [37]’s “Guidance on the conduct of narrative synthesis in systematic reviews” (Appendix B).

#### Braun and Clarke [38]’s thematic analysis

Using Braun and Clarke [38]’s approach to thematic analysis, the second team ‘actively’ read the included articles to find meaning and patterns in the data [39–43]. In phase two, ‘codes’ were constructed from the ‘surface’ meaning for the first twenty included articles. These codes were then collated and agreed upon and used to create a codebook. The codebook was used to code and analyse the rest of the articles using an iterative step-by-step process [44]. As new codes emerged, these were associated with previous codes and concepts. In phase three, an inductive approach allowed themes to be “defined from the raw data without any predetermined classification” [42]. In phase four, the research team discussed their independent findings and employed the “negotiated consensual validation” [34] to determine the final list of themes.

#### Hsieh and Shannon [45]’s directed content analysis.

The third team employed Hsieh and Shannon [45]’s approach to directed content analysis to “identify and operationalize a priori coding categories” [45–50] from Cruess, Cruess [51]’s “A schematic representation of the professional identity formation and socialization of medical students and residents: a guide for medical educators”, Barnhoorn, Houtlosser [52]’s “A practical framework for remediating unprofessional behavior and for developing professionalism competencies and a professional identity”, and Krishna and Alsuwaigh [53]’s “Understanding the fluid nature of personhood—the ring theory of personhood”. Any data not captured by these codes were assigned a new code [54]. The choice of articles was determined by the expert team and following discussion with independent experts. These articles are seen to best capture the breadth of thinking on the subject. ‘Negotiated consensual validation’ was used to achieve consensus on the final categories [55].

### Stage 3 of SEBA: Jigsaw Perspective

The Jigsaw Perspective employs Phases 4 to 6 of France, Wells [56]’s adaptation of Noblit and Hare [57]’s seven phases of meta-ethnographic approach to view the themes and categories identified in the Split Approach as pieces of a jigsaw puzzle. Overlapping or complementary elements within themes and categories are combined to create a bigger piece of the puzzle referred to as themes/ categories.

### Stage 4 of SEBA: funnelling

Themes/categories were compared with tabulated summaries [56, 57], which also included quality appraisals using MERSQI and COREQ [58, 59] to ensure that the themes/categories best captured the key elements of the included articles. The domains created from this process form the basis of the discussion’s ‘line of argument’ in Stage 6 of SEBA.

## Results

**Fig. 2:**
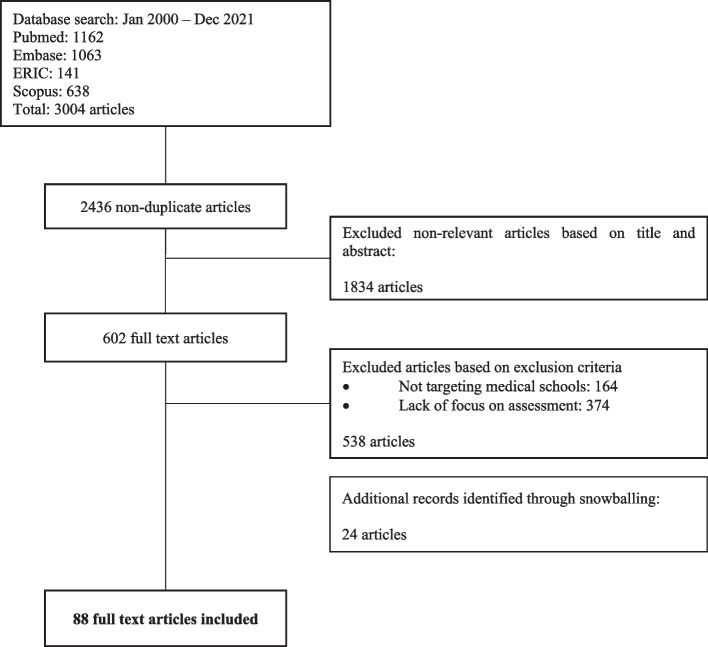
PRISMA Flow chart

Two thousand four hundred thirty six abstracts were reviewed, 602 full-text articles were evaluated, and 88 articles were included (Fig. 2). The domains identified were theories, assessments, and implementation. Much of the data were drawn from lists or were presented without accompanying explications. As such, we have summarised them in a series of tables for ease of review.

### Domain 1. Theories

Current assessment programs are shaped by the guiding PIF theory adopted. There were eleven theories of PIF employed by current assessment programs. We summarised these in Table 2.

**Table 2:** Theories guiding the assessment of PIF

Theories and Framework	Purpose/ Description
Kegan’s constructive development theory [60]	Kegan outlines 6 stages in cognitive development which affects all emotional and relational functioning: Stage 0 (incorporative balance): “in which reflexes are primary”, Stage 1 (impulsive balance): “in which knowing is only about one’s own immediate impulses”, Stage 2 (imperial balance): “in which the individual is aware of concrete and durable categories, that is, her or his own experiences as well as others’ experiences”, Stage 3 (interpersonal balance): “in which abstractions and more mutual relationships become possible”, Stage 4 (institutional balance): “in which understanding of systems, greater autonomy, and self- authorship become possible”, and Stage 5 (interindividual balance): “in which people become the directors and creators of systems, understanding how systems fit together meaningfully”
Pratt’s theory on professional identity formation [61]	This theory presents PIF as a process of interlinked work and identity cycles, in which identity construction is triggered by work-identity integrity violations which are resolved through 3 identity customization processes – enriching, patching and splinting A work-identity integrity violation occurs when there is a mismatch between what the individual is doing and what they believe they should be doing in keeping with their professional identity. In individuals with low job discretion and well-developed identities, minor integrity violations are likely to result in identity enrichment. On the other hand, in individuals with low job discretion and major identity violations, patching and splinting occur. In “patching”, an ‘ideal’ identity is adopted used to fill in the deficiencies in the individual’s professional identity, whereas in “splinting”, a former identity is used to protect the currently fragile professional identity
Wald’s theory on professional identity formation [62]	PIF is “an active, developmental process which is dynamic and constructive and is an essential complement to competency-based education” PIF “encompasses development of professional values, moral principles, actions, aspirations, and ongoing self-reflection on the identity of the individual and is described ultimately as a complex structure that an individual uses to link motivations and competencies to a chosen career role.” PIF involves “deepening of one’s commitment to the values and dispositions of the profession into habits of mind and heart” and is fundamentally ethical (including an ethic of caring) with development of a set of internal standards or an “internal compass” regulating professionals’ work Key drivers of PIF “include experiential and reflective processes, guided reflection, formative feedback, use of personal narratives, integral role of relationships and role models, and candid discussion within a safe community of learners (an “authentic community”)” Three central themes support and reciprocally enhance PIF • Reflective practice: self assessment of values, attitudes, beliefs, reactions to experiences and learning and experiential learning nurture PIF • Relationships: dependent on context and a collaborative environment, mentorship and role modelling, small group collaborative reflection and feedback, peer mentoring, interprofessional work, meaning making and group negotiation • Resilience: responding to stress in a healthy way with ‘bouncing back’ after challenges and growing stronger
Holden’s longitudinal framework through TIME [63]	This framework characterizes the physician identity according to 6 domains which further branch into 30 subdomains. The 6 domains are: attitudes, personal characteristics, duties and responsibilities, habits, relationships, perception and recognition The framework is mapped onto the 3 developmental phases of medical education (the undergraduate student, the clerkship-level medical student, and the graduating medical student), providing strategies for the longitudinal assessment and promotion of each subdomain at each phase [63]
Korthagen’s level of change model [64]	• The onion model describes 6 different levels on which reflection takes place, including environment, behavior, competencies, beliefs, identity, and, at the model’s center, mission • Core reflection ◦ Reflection on the level of mission is “concerned with what inspires us, and what gives meaning and significance to our work or our lives”. This is a transpersonal level, involving becoming aware of the meaning of our existence in the world and the role we see ourselves in ◦ Reflection on the level of identity, on the other hand, is about “how we experience ourselves and our self-concept” • The inner levels determines how an individual functions on the outer levels and vice versa • The model shows that aside from behaviour or competencies, there are also other essential qualities of a good teacher
Barnhoorn’s multi-level professionalism framework [53]	Adapted from Korthagen’s level of change model, this framework delineates 6 levels of influences at which the remediation of unprofessional behavior and development of professional identity can occur. These levels are: environment, behaviour, competencies, beliefs and values, identity, mission • Environment: “the diverse contexts in which the medical student lives, works and learns, and which influence his or her behavior” • Behaviour: the student’s performance which can be directly observed and assessed • Competencies: the integrated body of knowledge and skills that allows for professional behaviour • Beliefs and values: “the conceptions and convictions a medical student holds true regarding the medical profession and his or her place in it” • Identity: “the way one defines oneself in terms of characteristics, values, and norms, including the characteristics, values, and norms of the profession” • Mission: “the role the medical student sees for him- or her-self in relation to others”
Goldie’s social psychological levels of analysis [18]	Goldie’s social psychological levels of analysis builds on the Personality and Social Structure Perspective (PSSP) model, involving the application of identity formation and identity maintenance process It classifies medical student’s identity at 3 different levels: ego identity, personal identity, social identity, looking at the interplay between these levels • Ego identity: “the more fundamental subjective sense of continuity characteristic of the personality” • Personal identity: “at this level students find a fit between their social identity as ‘medical student’ and the uniqueness and idiosyncrasies of their learning/life history” • Social identity: “at this level, the student is most influenced by the pressure to fit into the available identity ‘moulds’ created by cultural and role-related pressures”
Jarvis-Sellinger’s conceptual framework of professional identity formation [65]	A model of action, based on grounded theory, that illustrates how the interactions between context, focus and catalyst aid medical students in processing their emerging professional identities Within this framework, context refers to the “details medical students use to describe an encounter or activity that has provoked reflection”; focus refers to what the medical students pay attention to in the encounter or activity; catalyst refers to a stimulus such as a learning event that triggers conscious thinking about professional identity within a specific context; while the process “signifies the ways in which medical students experience and describe navigating or negotiating their own emerging professional identities” Students’ reflections were noted to be focused on either their current identity (being) or their future identity (becoming)
Cruess et al.’s schematic representations of professional identity formation and socialization [52]	Within Cruess et al.’s schematic representation of professional identity formation, individuals enter medical school with their own identities and through a process of socialization, emerge with both personal and professional identities. The process of socialization involves individuals moving from legitimate peripheral participation in a community to full participation, primarily through social interaction. Socialization is influenced by multiple factors including the healthcare system; learning environment; role models and mentors; clinical and non-clinical experiences; self-assessment; formal teaching and assessment; symbols and rituals; family and friends; attitudes of patients, peers, health care professionals and the public; and isolation from peers
Hilton and Slotnick’s theory of “proto-professionalism” [66]	The authors propose a broad view of professionalism involving 6 domains, which include areas focusing on doctors alone (ethical practice, reflection and responsibility), and areas requiring collaboration (respect for patients, teamwork and social responsibility). The authors also coin the term “proto-professionalism” to describe the period of learning, experience and maturation to attain professionalism
Krishna et al.’s Ring Theory of Personhood [54]	• Assumes that PIF is part of an individual’s self-concept of personal identity • Suggests that identity can be captured by understanding conceptions of personhood • Identity creates values, beliefs and principles that determine thinking, decision making, conduct and action • The values, beliefs and principles must adapt to new settings, circumstances and these changes result in evolution in identity • Based on the Ring Theory of Personhood that there are 4 elements of identity corresponding to the 4 domains of personhood • The Innate identity draws on Innate Personhood. The Innate Ring is anchored in the belief that all humans are deserving of personhood, “irrespective of clinical status, culture, creed, gender, sexual orientation, religion, or appearance”. The Innate Ring contains gender, name, family identity, religious and cultural, community and nationality-based beliefs, moral values, ethical principles, familial mores, cultural norms, attitudes, thoughts, decisional preferences, roles, and responsibilities (henceforth beliefs, values and principles). These religious, cultural and societal inspired beliefs, values and principles can come into conflict with professional principles and values particularly when contending with withholding and withdrawing treatment [67], care determinations [68], collusion [69], and end-of-life care [70] often tread on Confucian-inspired beliefs [71] • The Individual Ring contains the unique characteristics and conscious function of the individual [72]. The identity associated with the Individual Ring is informed by the individual’s preferences, biases, beliefs, mores, norms, values and principles and the beliefs, values, and principles of the other rings. Balancing these sometimes-competing considerations in the face of a variety of psychoemotional, experiential, perceptual, and contextual considerations; individual preferences and decision-making styles and biases; and prevailing professional, sociocultural, legal, ethical, and personal considerations can result in dissonance between the different aspect of a medical student’s identity • The Relational Ring consists of relationships the individual holds to be important. These may come into conflict with legal, ethical, institutional, professional and societal values, beliefs considerations contained in the Societal Ring [73–76] • When the beliefs, values and principles being instilled are in conflict with those in one of the rings (disharmony) or between the rings of the RToP (dyssynchrony)

Of these eleven theories, there are three theories that have been especially influential to practice. One, Cruess et al.’s schematic representations of PIF and socialization [51] which touch on the notion that Oncology and Palliative Medicine postings may be both a Community of Practice (CoP) and a mechanism that facilitates the inculcation of the values, beliefs and principles and identity of the CoP or Socialisation process. Barab et al. [77] define CoPs as “a persistent, sustaining social network of individuals who share and develop an overlapping knowledge base, set of beliefs, values, history and experiences focused on a common practice and/or enterprise”. Cruess, Cruess [78] define socialisation as “a representation of self, achieved in stages over time during which the characteristics, values, and norms of the medical profession are internalised, resulting in an individual thinking, acting and feeling like a physician”. The socialization process in turn is reliant upon an organised, supportive sense of community, highlighting the entwined relationships between CoPs and the socialisation process. The socialisation process is also dependent upon the CoP’s provision of personalised, timely, appropriate, holistic and longitudinal support through a combination of mentoring, role modelling, coaching, networking, advicing, supervision, tutoring, feedback, guided reflections and supervised experiential learning within a safe and structured learning environment.

Two, underscoring the interrelated nature of environmental factors and identity formation is Barnhoorn’s multi-level professionalism framework [52] that builds on Korthagen’s level of change model [79]. These theories invite the notion of environmental factors within a CoP influencing the medical student’s current beliefs, values, competencies and their identities and behaviour.

Three, Krishna’s Ring Theory of Personhood (RToP) builds on links between identity, mission and behaviour, and the impact of environmental factors on PIF (Fig. 1). The RToP also draws on Wald’s [80] and Pratt’s [81] theories on PIF to explicate the ties between the environment, experience and PIF. The RToP suggests that the medical student’s current beliefs, values, competencies and regnant identities and behaviour are shaped by their self-concepts of personhood or “what makes you, you”. Specifically, the RToP posits that the medical student’s current beliefs, values, and competencies (henceforth belief system) within the medical student’s Innate, Individual, Relational and Societal domains of personhoods shape their corresponding identities. Changes in the belief system in each domain, result in changes in their corresponding identities.

**Fig. 3:**
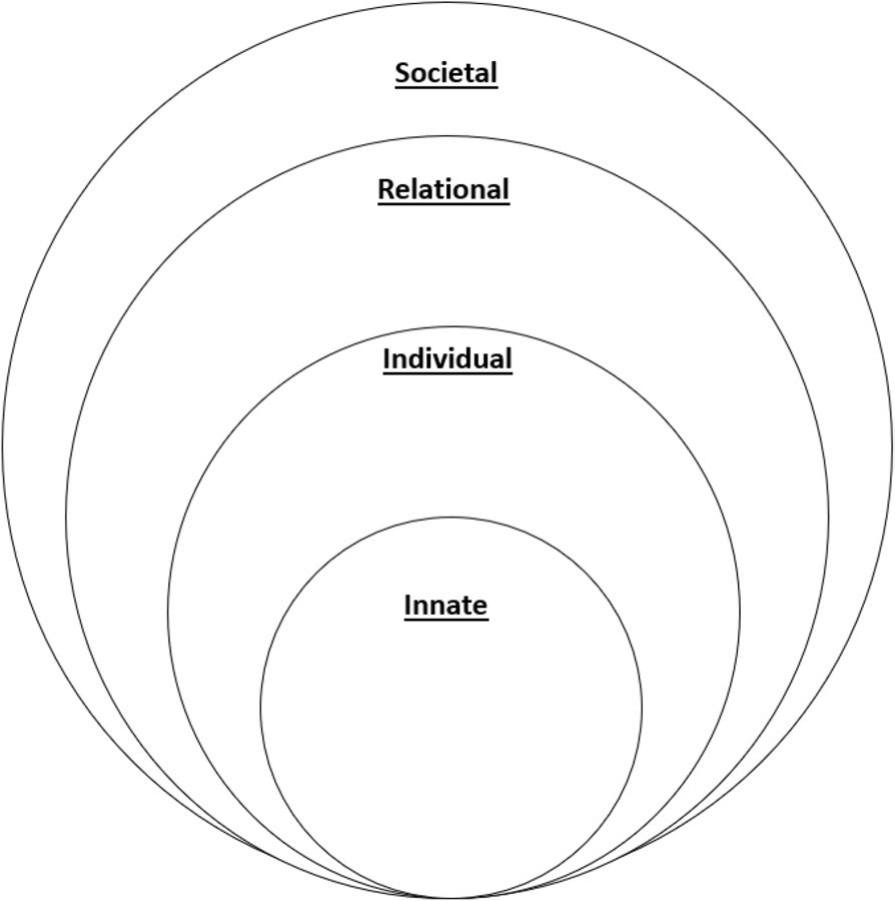
The Ring Theory of Personhood

The RToP postulates that the Innate, Individual, Relational and Societal domains of personhood which may be represented as intertwined rings (Fig. 3). The Innate Identity is derived from regnant spiritual, religious, theist moral and ethical values, beliefs, and principles within the Innate Ring. The Individual Ring’s regnant belief system is derived from the medical student’s thoughts, conduct, biases, narratives, personality, decision-making processes, and other facets of conscious function which together inform the medical student’s Individual Identity. The Relational Identity is born of a belief system derived from the values, beliefs and principles governing the medical student’s personal and important relationships. The Societal Identity is shaped by regnant societal, religious, professional, and legal expectations, values, beliefs, and principles which inform their interactions with colleagues and acquaintances.

Growing clinical, mentoring, research and personal experiences, reflections, and competencies as the medical student progresses through the Oncology and Palliative Care posting, result in changes in the belief systems and with that, changes to their corresponding identities. However, the nature of the changes in identity is determined by three considerations. One, when practical, environmental, and professional influences are consistent with the medical student’s belief system, there is ‘resonance’. When resonant values, beliefs and principles are adapted and reprioritised to better fit current practice, there is ‘synchrony’. Conversely, when there is conflict between the introduced and remnant beliefs systems in one ring, there is ‘disharmony’. Should the conflicts extend to more than one ring, there is ‘dyssynchrony’.

Two, how synchrony, resonance, disharmony, and or dyssynchrony are attended to is shaped by the medical student’s psychoemotional state, circumstances, goals, experiences, motivations, enthusiasm, idealism, abilities, competencies, virtues, expectations, knowledge, skills, and attitudes (henceforth narrative). It is also influenced by their contextual considerations including prevailing research, clinical, and organisational considerations, professional codes of conduct, societal expectations, ethical and legal standards (henceforth contextual considerations).

Three, ‘Identity work’ involves addressing the presence of synchrony, resonance, disharmony, and or dyssynchrony, in a manner that is consistent and appropriate to the medical student’s narratives, contextual considerations and available support and guidance. Pratt’s theory on PIF [81] is employed to ‘identity work’ using patching and splinting. In “patching”, the medical student adopts their perception of an ‘ideal’ identity to fill in the deficiencies in their professional identity, whereas in “splinting”, the medical student employs a former identity to protect the currently fragile professional identity. The concept of ‘identity work’ also encapsulates the decision not to act.

### Domain 2. Assessment

#### Principles, information considered and methods of assessments

Governed by their respective guiding theories, current assessment factors and methods are summarised in Table 3.

**Table 3:** Principles, Information considered and methods of assessments

Ethical Framework		Number of articles employing it and references in brackets
Korthagen’s level of change model		One [53]
Barnhoorn’s multi-level professionalism framework		One [78],
Goldie’s social psychological levels of analysis		Eight [52, 53, 62, 79–83]
Kegan’s constructive development theory		Four [63, 80, 81, 83]
Pratt’s theory on professional identity formation		Three [18, 62, 63]
Wald’s theory on professional identity formation		Five [65, 81, 84–86]
Cruess et al.’s schematic representations of professional identity formation and socialization		Nine [19, 53, 62, 84, 87–91]
Krishna’s Ring Theory of Personhood		Three [13, 17, 28]
Principles	Information considered (in the context of theories)	Methods of assessment
longitudinal assessments [5, 29, 63, 78, 83, 85, 88, 90, 92–103]	Personal [13, 17, 19, 28, 52, 53, 60, 65, 66, 78, 80, 81, 83, 88, 89, 91], practical [13, 66, 78, 88], clinical [17, 28, 52, 60, 65, 81, 82, 87–89, 91], environmental [13, 17, 18, 28, 52, 58, 60, 63, 65, 78, 81–83, 87], academic [17, 81], research [17, 61], systems-based considerations [13, 60, 81, 87, 88, 90];	summative assessments [104],
multidimensional approach [87, 97, 104, 105]	the medical student’s social [28, 60, 65, 66, 79], personal [13, 17–19, 28, 52–54, 63, 66, 81, 87, 89], demographic, contextual, academic, research, clinical, and professional values [19, 62, 63, 65, 66, 78–81, 83, 84, 87–91], their **beliefs** [13, 17, 18, 28, 52–54, 60–62, 65, 78, 80–83], **principles** [28, 52, 60, 62, 65, 80, 87, 89], **experiences** [13, 17, 18, 28, 52, 54, 60–63, 65, 66, 78, 80–82, 89–91], **competencies** [19, 52, 53, 62, 63, 66, 79, 81, 84, 87–89], and **goals** [13, 17, 19, 28, 52, 54, 81, 82, 89, 90]	formative assessments [63, 87, 88, 96]
multimodal approach to assessing PIF [5, 19, 29, 63, 87, 91, 95, 96, 100, 104, 106–111]	environmental conditions, the requirements [87], and influences [62, 66, 83, 89] within the practice setting	use of mixed methods [19, 29, 78, 80, 100, 103, 106, 107, 112–114]
site-specific assessments [107, 112, 115]	the impact of the formal [18, 52, 53, 65, 78, 81, 83, 87], informal [18, 65, 78, 81–83, 87], and hidden curriculum [18, 53, 65, 66, 78, 80, 81, 83, 87]	
assessments at multiple time points [80, 83, 85, 92, 107, 112, 113]	the program and practice expectations [87, 88] on conduct, competencies, attitudes, and goal [13, 52]	
use of multiple assessors [29, 81, 85, 87, 100, 104, 106, 107, 110–113, 116, 117]	the medical student’s ethical position [63, 81, 100, 109, 112, 114, 116, 118–121]	
	The medical student’s moral position [80, 84, 87, 95, 100, 114, 118, 119]	
	The medical student’s professional position [53, 63, 80, 81, 90, 91, 98–102, 106, 108, 109, 111, 112, 114, 122]	
	medical student’s values, beliefs and principles - If specific to med student: 17, 18, 19, 28, 52, 53, 54, 61, 62, 63, 64, 66, 67, 68, 69, 72, 73, 74, 76, 77, 81 -If not: 78, 79, 13	
	The medical student’s actions, attitudes [63], conduct, reflective practice [63] and support mechanisms [63] over time	
	the demographical [91], historical [83], experiential [63, 90] and environmental factors [17, 18, 52, 53, 63, 65, 81, 83, 90] influencing concepts of identity	

#### Existing modalities of PIF assessment

Most evaluations focus upon character traits, conduct and the cognitive base. Whilst we discuss the key approaches, we summarise the tools identified in Table 4

**Table 4:** Tools used in the assessment of PIF

Tools of assessment
- Guided feedback [101, 122] - Questionnaires [116] - Structured activity—“A Learning Experience” [116] - Brown’s Guide/ BEGAN tool (the Brown Educational Guide to the Analysis of Narrative) [101] - Reflection Evaluation for Enhanced Competences Tool Rubric (REFLECT) [86, 92, 95, 123] - Thematic scoring (“to map and grade the reflection themes”) [95] - Self-reflection and insight scale (SIRS) [115] - Groningen Reflection Ability Scale (GRAS) [115, 124] - Reflective ability rubric [114] - Reflection-in-Learning Scale [116] - Self-assessment [87] - Professional self-identity questionnaire (PSIQ) [105] - Moral reasoning assessment [87] - Observations during clinical assessments [110] - assessment of learning environments [110] - Mentor facilitated conversations [110] - Professional identity essay [80, 119, 120] - Stage-specific attribute scales (SASs) [91] - Physician professional identity survey [97] - Identity integration (IdIn) survey [97] - Developing Scale [84, 91] - Professional identity questionnaire (PIQ) [125]

##### Reflections (11/88, 12.5%)

Reflective practice plays a critical role in PIF [82, 83]. Current assessments of reflections are guided by Kegan’s constructive-developmental theory [82], Gibb’s reflective circle [62], Schon’s theory of the reflective practitioner [63], or Boud’s models of reflective thinking [84].

##### Guided Feedback (8/88, 9.1%)

Guided, multi-sourced [82, 85, 86] and competency-based [65] feedback influence the socialization process [65, 87], guides role modelling of good behaviour [88], shapes critical thinking [89], addresses bad professional behaviour [88], reinforces commitment to the professional role [90] and builds emotional resilience [89].

##### Portfolio (11/88, 12.5%)

Portfolios serve as a powerful tool for reflection and reinforce professional and organizational values [91]. To achieve these objectives, portfolio includes a mix of generalised assessment methods [28], longitudinal assessment data [60, 61, 82], self-assessments [60] of work and accomplishments [91] and reflections [28, 60, 64, 66, 84, 91] on ethical issues [66], professional breaches [66], and moral dilemmas [66]. Other contents of portfolios are highlighted in Table 5.

**Table 5:** Contents of portfolio

Portfolio contents
- Material relevant to the roles of the healer and professional [99] - Autobiography [100] - Individual Hippocratic oath document [100] - Health contract [100] - Myers-Briggs personality inventory [100] - Self-grading professional development [126] - Peer feedback [126] - Reflective writing [98, 100, 121, 126] - Essays on physician–patient Relationship [126] - Comment cards [126] - Volunteering experiences [63, 126] - Elective materials [126] - Documentation of other assessments from faculty and peers [126] - Evaluations of standardized patient interactions [126]

#### Tools contextualised to the nature of PIF

Assessments of PIF are often stage-based.

##### Stage-based nature of PIF

**Fig. 4:**
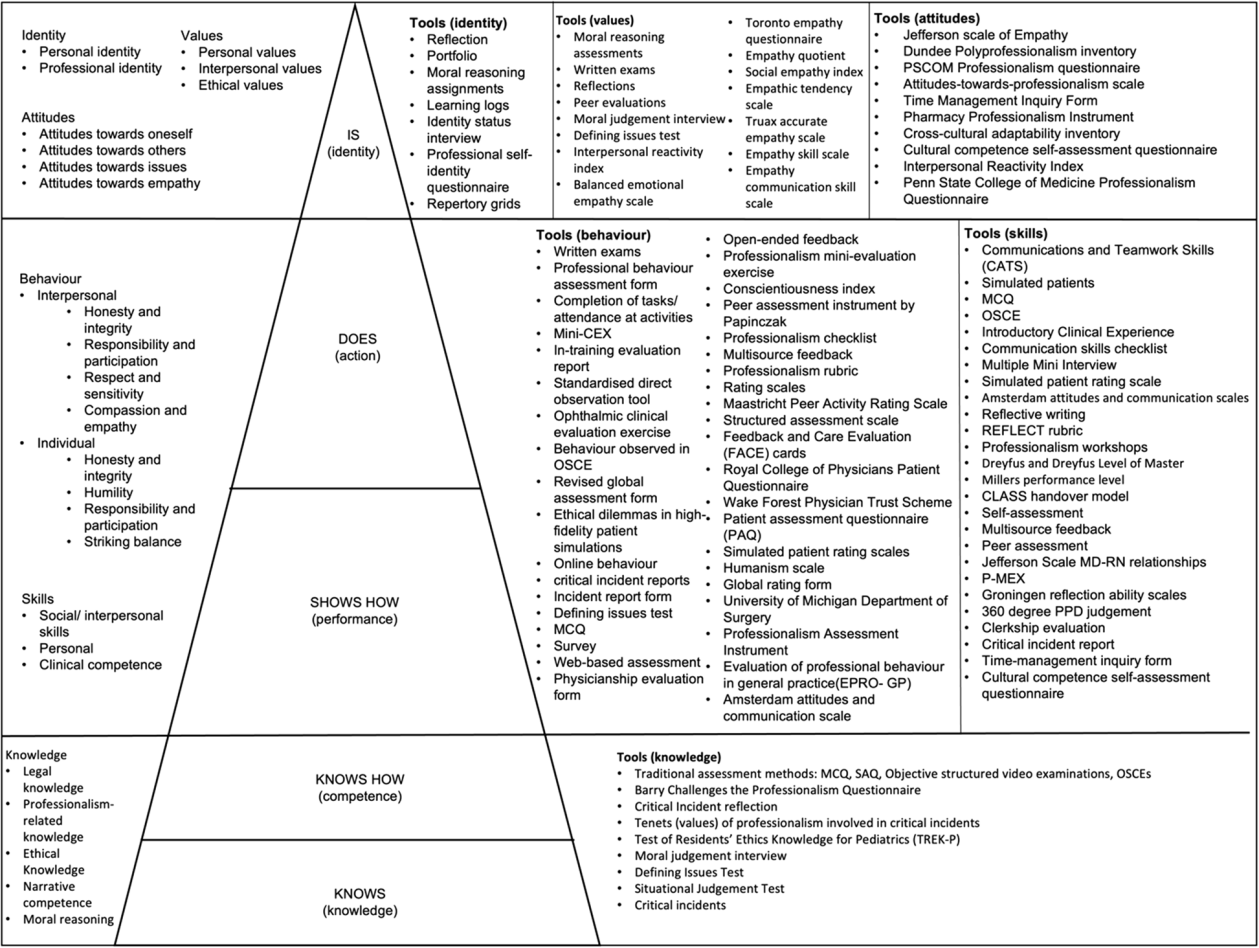
Tools used in the assessment of PIF grouped according to the stages of Miller’s pyramid

Assessments often reflect the notion of a stage-specific development of PIF exemplified by Cruess, Cruess [17]’s amendment of Miller’s pyramid. Figure 4 stratifies the assessment tools in accordance with these stages. The included articles for each stage are also found in Appendix C.

**“Knows”/ Knows How” (10/88, 11.4%)**

‘Know’ considers the medical student’s level of knowledge [17]. ‘Know how’ determines if the medical student knows how to analyse, interpret, synthesise, and apply the knowledge [17]. These elements inform behaviour [52], and reflect attitudes [67].

**“Shows how”/ “Does” (34/88, 38.6%)**

“Shows how” considers the demonstration of skills and interpersonal and individual behaviour [17]. Behaviour is assessed by teachers/ tutors [68–70], supervising physicians [65, 70], patients [66], peers [69–71, 82, 87, 88] and or may be self-assessed [70]. However, it is peer assessments that are considered the most reliable and authentic means of assessing this aspect [88].

Assessment of the “Does” aspect involves the demonstration of skill and behaviour even when not being formally assessed [17]. This aspect too is best reflected in peer assessments [17]. Skills are assessed by supervising physicians [65] or peers [65, 71, 82], using workplace-based assessments (e.g. actual patient encounters [70], simulation (e.g. simulated patients [70]) and OSCEs [62, 71, 72, 82].

**“Is” (18/88, 20.5%)**

At the “Is” stage, self-concepts of identity are evaluated by questionnaires [73, 74], and moral reasoning [75].

We summarise the prevailing tools in Table 6.

**Table 6:** Articles referencing the self-concepts of identity

	Number of articles (88)	References
Beliefs	3	[91, 99, 119]
Mission	0	NA
Abilities/ experiences	23	[79–82, 85, 94, 95, 99–101, 103, 105, 111, 113, 114, 116, 117, 120, 122, 123, 127–129]
Behaviour	22	[79, 80, 82, 83, 91, 92, 94, 98–100, 102, 106, 108, 109, 111, 113, 115, 116, 127–130]
“Knows”/ “Knows how”	10	[19, 53, 63, 95, 104, 112, 113, 120, 121, 126]
“Shows how”/ “Does”	34	[19, 29, 63, 78, 79, 84, 87, 88, 92–95, 99, 100, 102–104, 106–109, 111, 113, 117, 120, 121, 126–133]
“Is”	18	[5, 29, 63, 80, 84, 87, 94, 99, 104, 107, 109, 113, 120, 121, 133–136]
Reflections	11	[63, 86, 92, 95, 101, 114–116, 122–124]
Guided feedback	8	[63, 80, 100, 102, 109, 111, 122, 127]
Longitudinal assessments/ portfolio	11	[29, 63, 98–100, 104, 110, 116, 121, 126, 128]
Host organization	4	[88, 103, 127, 129]
Socialisation	13	[63, 80, 81, 86, 90, 98, 100, 101, 104, 114–116, 126]
Community of Practice	6	[63, 88, 100, 103, 111, 112]
Remediation	11	[78, 87, 89, 103, 107, 115, 118, 126, 129, 132, 137]
Longitudinal assessments	20	[5, 29, 63, 78, 83, 85, 88, 90, 92–103]
Holistic/ multidimensional assessments	4	[87, 97, 104, 105]
Multimodal assessments	16	[5, 19, 29, 63, 87, 91, 95, 96, 100, 104, 106–111]
Site-specific assessments	3	[107, 112, 115]
Multiple timepoints	7	[80, 83, 85, 92, 107, 112, 113]
Multiple assessors	14	[29, 81, 85, 87, 100, 104, 106, 107, 110–113, 116, 117]

##### Assessing socialization (13/88, 14.8%)

Socialization can be assessed through reflections [76, 83, 84, 87, 90–96], longitudinal assessments in portfolios [60, 76, 82, 87, 91], and guided feedback.

### Domain 3. Implementation

#### Who assesses PIF?

PIF can be self-evaluated, or assessed by peers [87], faculty [87, 91, 97], clinical supervisors [98], patients [98] and other healthcare professionals [98, 99]. Elliot et al. [87], suggests that peer assessments are a reliable evaluation method, particularly in a group setting or when involving facets of communications, self-awareness, self-care and growth. Peer assessments, however, are vulnerable to misrepresentation and collusive agreements [100]. Self-assessments are useful, particularly when triangulated with other source material [97]. Van Mook et al. [97] suggests that faculty assessments are dependent on individual attitudes, motivation and instructional skills of faculty members.

#### Administrative Considerations/Role of host organisation (4/88, 4.5%)

Assessments should be explicit, confidential [88], and accompanied by accessible feedback and results [101, 102]. These considerations underline the importance of faculty training and emphasize the need for clearly stipulated assessment guidelines to safeguard fair assessments [69, 92].

#### Remediation (11/88, 12.5%)

Remediation is increasingly acknowledged as an elemental aspect of any training process that must be informed by a thorough assessment [75, 98]. To remain transparent, confidential and personalised, the remediation programme must be robust and longitudinal, involving goal setting [103], provide feedback [104] evidence of the completion of modules [75, 103], and the inclusion of reflections [75, 98, 103]. Remedial processes should be supported by accessible mentoring, coaching and counselling support [69, 75, 96, 101] and personalised and timely follow-up and monitoring of progress [105].

The procedures including warnings [105, 106], suspension, dismissal/ expulsion [106, 107] after appropriate discussions and escalation to senior faculty, dean’s office and directors [91, 98, 106], must be clearly delineated.

### Stage 5 of SEBA: analysis of evidence-based and non-data driven literature

The themes drawn from evidenced-based publications were compared with those from non-data-based articles (grey literature, opinion, perspectives, editorial, letters). The themes from both groups were similar and non-data-based articles did not bias the analysis untowardly.

#### The reiterative stage of SEBA

As part of the SEBA process, the expert team evaluated the initial data found in this review. This process drew attention to several recent reviews that were subsequently considered in the analysis of the data to ensure an evidence-based, clinically relevant synthesis of the data.

Recent reviews on the use of portfolios [108] amongst medical students as a means of mapping longitudinal development and identity formation underscore the key elements within a portfolio and highlight the efficacy of e-portfolios.

Similarly, reviews of how medical students, physicians and nurses caring for dying patients [13], coped and its impact on their PIF suggest that Krishna et al. (2020)’s [53] Ring Theory of Personhood (RToP) provides an effective means of capturing changes in identity over time and in the face of crises. The RToP was subsequently added to the list of current guiding theories (Table 3). These insights helped frame the discussion.

## Discussion

### Stage 6 of SEBA: synthesis of SSR in SEBA

**Fig. 5:**
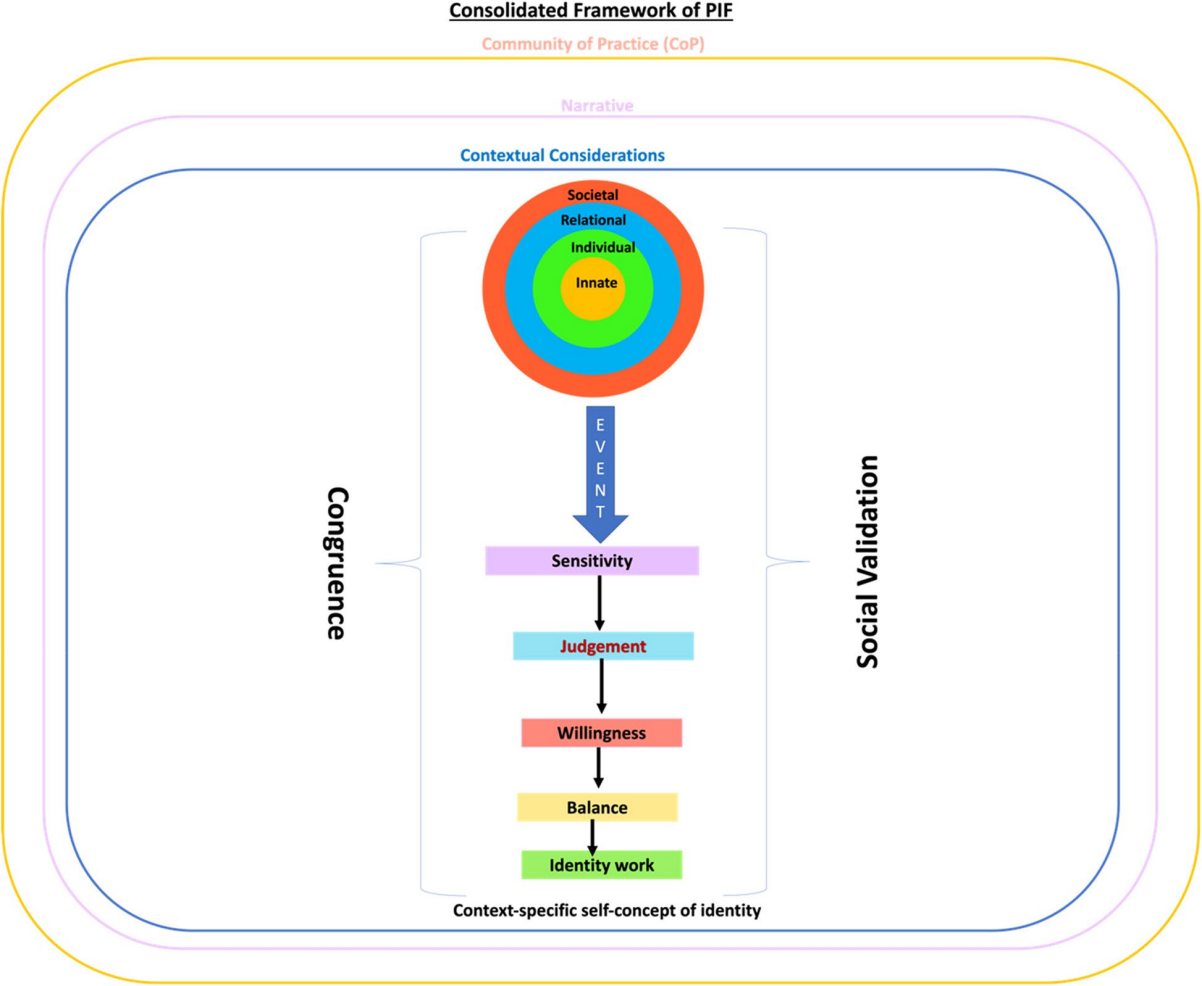
Krishna-Pisupati model

To answer its primary research question which was “What is known about assessments of PIF amongst medical students?”, this SSR in SEBA forwards the Krishna-Pisupati’s model (Fig. 5) of current concepts of PIF amongst medical students. Drawn from included articles and based on regnant theories and practices, the Krishna-Pisupati’s model provides insights into the rationale for the various assessments employed to direct timely support of PIF amongst medical students.

Summarising and contextualising the findings of this SSR in SEBA, the Krishna-Pisupati’s model is constructed around the key themes of leading PIF theories. These are the structural and individual aspects of PIF theories. Simply put, the structural aspects may be encapsulated by the notion of Oncology and Palliative Care postings functioning as CoPs. The individual aspects on the other hand highlight the role each medical students plays in shaping their professional identity.

#### Structural

The notion that Oncology and Palliative Care postings may be considered as CoPs, pivots on three postulations. One, Oncology and Palliative Care postings offer a safe and structured learning, organizational, professional, clinical, practice, academic and research environment for medical students (henceforth learning environment). The notion of the interactions extending beyond the dyadic relationship between tutor and student and embracing the influences of the learning structure and culture, resonates with Korthagen’s level of change model [79] and Barnhoorn’s multi-level professionalism framework [52].

Two, the structure afforded Oncology and Palliative Care postings with clearly established roles, responsibilities, codes of practice and expectations, clearly signposted trajectories, training methods, communication processes, interactive and context-rich content, expectations, assessments, and support mechanisms. The structure provides guided immersion into the Oncology and Palliative Care work environment and mentored progress; from a peripheral role in the team to being a part of the care team which are at the heart of CoPs. It is also supported by the provision of timely and personalised coaching, counselling, supervision, role modelling, mentoring and guided reflection (henceforth mentoring umbrella), an inherent aspect [109] of an Oncology and Palliative Care posting as an interactive developmental process.

Three, CoPs also structure effective evaluation, longitudinal support and follow-up of the medical student. This holistic and longitudinal assessment process considers the medical student’s narrative, their contextual considerations, self-awareness, and willingness to engage. Also integrated into guiding assessments aimed at directing timely and personalised support, within this personalised developmental process are the medical student’s maturing cognition, developing resilience and competencies, emotional growth, changing narratives, evolving character and motivations, growing ability to engage support networks, participation in reflective practices and seeking support and guidance and in taking on feedback. This highlights the importance of the mentoring umbrella [110] and perhaps just as significantly, the need for faculty training and support to apply the appropriate assessments and provide timely, personalised, and appropriate feedback and support.

The combination of a mapped training program, the mentoring umbrella, trained faculty, structured assessments, guided reflections and supervised experiential learning within the curated learning environment aspects support a structured, and personalised Socialisation Process. The Socialisation Process also thrives in the presence of a congruence between the medical student’s characteristics, goals, expectations, and stages of development and the training and experiences being offered in the Oncology and Palliative Care postings. Peh et al. [109] noted that when pitched at a level appropriate for their training, competencies and experience, medical students better relate to the value of Palliative Care postings and recognise the experiences afforded (Congruence). Similarly, the content, standards, roles and responsibilities expected of medical students and the approaches used to train and support the medical student must be consistent with the practice settings, program culture and academic structure as well as regnant sociocultural considerations (Social Validation).

#### Individual influences

The individual aspects of current PIF theories are virtually encapsulated by a combination of the RToP and Wald’s [80] and Pratt’s [81] theories on PIF. The RToP sketches the influence of the prevailing belief system, the particular medical student’s narratives, contextual and environmental considerations and their developing competencies on their maturing self-concept of identity and personhood. Wald’s [80] and Pratt’s [81] theories on PIF offers to explain the consequent changes in the medical student’s RToP during their Oncology and Palliative Care postings [11, 12, 111, 115]. Concurrently, the combination of the RToP and Wald’s [80] and Pratt’s [81] theories on PIF provide new insights into key elements of the CoP’s Socialisation Process.

The concept of ‘sensitivity’ describes the medical student’s awareness of resonance, synchrony, disharmony and or dyssynchrony when transitioning to different roles, responsibilities, practice settings, cultures, and structures and goals; and or exposure to death [112, 113] and dying [18, 20], moral distress [114, 116, 117] and the demands of dignity-based patient [118] centred care [119–128, 131, 138], ubiquitous with Oncology and Palliative Care postings [11, 12, 111, 115] (henceforth events). The medical student’s ‘judgement’ attributes significance to these ‘events’. ‘Judgement’ determines if the event has resulted in resonance, synchrony, disharmony and or dyssynchrony and if these effects warrant attention. “Willingness’ refers to the motivation of the medical student to attend to the effects of events and the resonance, synchrony, disharmony and or dyssynchrony caused. With events likely to cause a mix of resonance, synchrony, disharmony and or dyssynchrony, ‘balance’ sees that the medical student prioritise adaptations to maintain their overall identity. ‘Sensitivity’, ‘events’, “willingness’, ‘judgement’ and ‘balance’ are influenced by the medical student’s narratives, contextual considerations, competencies, stage of development and how their changing narratives, contextual and belief systems are supported.

Housed within a structured and supported CoP, Oncology and Palliative Care postings [11, 12, 111, 115] can modulate the influence of environmental and contextual considerations, and their effects upon ‘sensitivity’, ‘events’, “willingness’, ‘judgement’ and ‘balance’. It is also able to tailor timely, personalised, and appropriate support to attend to the medical student’s narratives, contextual considerations, competencies, and stage of development. The provision of support is also influenced by the CoP’s access to trained mentors who are able to offer timely, appropriate, personalised and holistic guidance and longitudinal support.

The support provided by the combination of a mapped training program, the mentoring umbrella, trained faculty, structured assessments, guided reflections and supervised experiential learning within the curated learning environment galvanises the medical student’s ‘willingness’ to address ‘events’ and the resultant is resonance, synchrony, disharmony and or dyssynchrony. This will encourage and assist efforts to carry out the required ‘identity work’. ‘Identity work’ refers to efforts by the medical student to adapt their identity to their current situation (Context-specific self-concept of identity). This may include ‘inaction’, ‘patching’, ‘splinting’ or ‘enriching’ which is the strengthening of the medical student’s current value system and reinforcement of their regnant professional identity [81]. Kuek et al. [27] and Ho et al. [25] suggest that if the medical student determines that their present identity fits the role, responsibility, circumstances, setting and or practice, there is synchrony, and the prevailing identity is ‘enriched’. If there are differences between the inculcated and regnant values, beliefs and principles that cannot be easily addressed, the medical student may adopt Pratt’s concepts of patching or splinting their current identity [81]. Lacking experience, knowledge, skills and the appropriate competencies, novices start by drawing on their previous identities to ‘splint’ their current identities [106]. As they develop experience, insights, and inculcate their reflections, the appropriate values, beliefs and principles, a medical student may sequester the identity of a ‘senior’ or experienced clinician to ‘patch’ their new identities as they grow and adapt to their new roles [81]. With greater experience, reflection, feedback, mentoring and role modelling, a customised sense of identity that is more robust is created [106]. Identity work on the customised self-concept of identity often simply involves ‘fine tuning’.

Appreciation of the cycle of ‘sensitivity’, ‘judgement’, ‘balance’, ‘willingness’, and the capacity and ability to carry out ‘identity work’ affords insights into the development of the medical student’s PIF. This is important as medical students move from an individualised sense of identity that is focused upon their own needs, to an interpersonal sense of identity that considers the needs of others and finally, to an institutional identity that considers, espouses and role models the program’s shared identity. These considerations underline the importance of faculty training and support within the CoP.

Based on the structural and individual considerations, Krishna-Pisupati’s model is presented as three concentric rings. The outer ring underlines the notion of Oncology and Palliative Care postings existing as CoPs. The second ring recognises the medical student’s narratives which influence the medical student’s ‘sensitivity’, ‘judgement’, ‘balance’, ‘willingness’, and capacity and ability to carry out ‘identity work’. The narratives and the structure of the CoP shape the lens through which the medical student interprets the contextual considerations represented by the third ring.

Krishna-Pisupati’s model maintains that assessments of PIF should be seen as a means of guiding medical students as they develop their PIF and as a means of directing timely and appropriate support to them when they need it. In using the Krishna-Pisupati model to address the secondary research question “What are the tools used to assess PIF in medical students?”, Table 7 reveals a wide variety of context-specific largely ‘unidimensional assessments’ focused on self-assessments, reflective practice, single time point identity assessments and competency evaluations.

**Table 7:** Tools used to assess PIF

Tools of assessment
- Self-assessment [87] - Moral reasoning assessment [87] - Observations during clinical assessments [110] - assessment of learning environments [110] - Mentor facilitated conversations [110] - Professional identity essay [80, 119, 120] - Stage-specific attribute scales (SASs) [91] - Physician professional identity survey [97] - Identity integration (IdIn) survey [97] - Developing Scale [84, 91] - Professional identity questionnaire (PIQ) [125] - Professional self-identity questionnaire (PSIQ) [105]

Similarly in answering the research question “What considerations impact the implementation of PIF assessment tools amongst medical students?” the Krishna-Pisupati model (Fig. 5) reveals the variability in who assesses, supports, and remediates PIF related issues. Aside from the variety of tools employed, there is also significant inconsistencies amongst current approaches adopted to assess self-concepts of identity.

Based on these findings, the Krishna-Pisupati model forwards a set of observations that could provide a framework for more effective assessments of PIF processes.

##### A framework for the assessment of PIF

The insights garnered from addressing its primary and secondary research questions highlight the need to underline the goals of assessments of PIF. This is to direct timely and personalised mentoring support, and appropriate feedback and guide longitudinal communication between the host organization, mentors and tutors and medical students in their Oncology and Palliative Care postings. The goal of this process is to nurture a balanced and effective professional identity.

We set out a framework to guide assessments of PIF.

One, the assessment of PIF must be a personalised process. A personalised assessment program [28] recognises the influence of the medical student’s pre-existing identities, personal histories, demographics, professional, social, personal, academic, research, clinical and practice circumstances which shape their beliefs, values and principles and inform their thinking, decision making, actions, conduct and clinical practice. These considerations represent the foundational aspects of PIF.

Two, an assessment program must adopt a multi-source [82] and multidimensional [134] assessment process involving a variety of tools. Recognizing the primary role of the assessment process is to provide holistic support mix of tools that will capture the inputs of patients [98], peers [87], tutors and the interprofessional team and is a practical solution for the absence of a singular tool to assess PIF. A mix of regnant tools also allows for a more considered assessment of the medical student’s progress, needs and identity formation. It also accounts for their individual levels of knowledge, skills, attitudes, and competencies; conduct, decision-making processes, interprofessional and collaborative practice; patient interactions; and their coping and needs. This personalised time-specific, context-dependent [68] evaluation also considers the practice environment and support mechanisms available to the medical student. This is especially important in Oncology and Palliative Medicine postings where medical students are especially affected by exposure to death and the care of dying patients (henceforth death and dying) [25].

Three, the program must be longitudinal [78, 82] and capable of capturing the medical student’s needs and progress along their development trajectories. As a result, assessments at different time points [78, 90, 130] are encouraged as are due consideration of the medical student’s reflections, perspectives, and positions on a variety of matters. This is especially important when experiences with death and dying in settings associated with Oncology and Palliative Medicine such as Intensive Care and Paediatrics are affected by growing experiences, resilience, knowledge, skills, attitudes, competencies, and insights [13–15, 20, 25, 132].

Four, the use of a variety of assessment methods, involving input from different assessors [75] at different time points [78, 90, 130] and setting in tandem with self-assessments, reflections, and feedback; underlines the need for the assessment program to be capable of contending with different data sources. This assessment process must also be able to blend the data to provide a sketch of the medical student’s progress at a specific time point. At the heart of this must be clarity on what is being assessed, particularly when these assessments can contain significant data.

Five, the assessment program must be accessible, robust [139] and institutionally supported [133]. Influenced by our recent review of portfolio use amongst medical students [108], this SSR in SEBA proposes the use of e-portfolios to enhance accessibility of the data. E-portfolios provide medical students with easy access to entries, promote reviewing and reflecting upon their experiences and feedback, make adding corroborative evidence from a variety of sources and formats including videos or website links easier, and facilitate the employ of multi-source and longitudinal assessments and feedback [129, 135, 137, 140, 141]. An e-portfolio’s accessibility also provides supervisors with easy access to holistic and longitudinal data to effectively appraise a medical student’s progress and coping, evaluate their personal needs, and provide timely and appropriate feedback and support [129, 135, 137, 140, 141].

Six, the inclusion of written, electronic, audio and video data, drawings, paintings, poetry, reflections, and assessments in e-portfolios underline the need for a structured approach [136, 142]. This structure is delineated by clearly stipulated learning objectives and contents, professional standards and expected roles and activities [129, 135, 137, 140, 141].

Seven, the structure of the e-portfolios must be sufficiently flexible [143–145] to contend with different practice settings and program goals; guide reflections and feedback, and include a “choice of materials by the student” [136] and “individualised selection of evidence” [146].

Eight, assessments of PIF must be overseen by the host organization. This is especially important given that assessments of PIF are carried out over time and in different settings involving different team members, peers, and tutors. As a result, team members, peers and tutors need to be trained to evaluate and even provide feedback in a personalised, appropriate, timely, specific manner. Those assessing PIF should exemplify the traits that they are assessing others for, and students should be safe-guarded from unfair evaluations [147].

Nine, students must see the value in these assessments [147] for there to be meaningful growth in PIF. There is an inherent risk of turning PIF into “deliverables” which can add further stress and anxiety to the process of PIF and cause genuine assessments to be masked by attempts to manage others’ impressions. PIF must be made an explicit goal of medical education with avenues for students to reach out for support.

**Future additions**

Considering the growing data on PIF, portfolio use and assessment methods, there are several potential facets that ought to be considered in the future including the employ of e-portfolio-based assessments, feedback, reflective and support mechanism to appraise and support PIF [144].

Ngiam et al. [20], Kuek et al. [27] and Ho et al. [25]’s reviews of how medical students and physicians cope with confronting death and caring for dying patients raise the notion of an RToP-based tool. Ngiam et al. [20], Kuek et al. [27] and Ho et al. [25] posit that a better understanding of changes in concepts of personhood will provide insights into the medical student’s evolving identity, and their decision making processes, thinking, actions, conduct and clinical practice. Furthermore, these authors suggest that an RToP based tool populated with data drawn from the e-portfolio or directly from the regular application of the RToP could provide insights into the medical student’s personal experiences, coping and needs. Such insights could help direct timely, personalised, and appropriate support to medical students in need.

Concurrently, Tan et al. [108]’s review of portfolio use amongst medical students suggests that the employ of Hong et al. [26]’s and Zhou et al. [5]’s adaptation of Norcini et al.’s concept of micro-credentialling and micro-certification in medical education [148] will allow benchmarking of their progress. Inspired by Wald et al.’s [80] notion that PIF is shaped by developing clinical competencies, ‘general micro-competencies’ facilitate appraisal of the medical student’s progress from one competency-based stage to another within the posting [149, 150]. Tan et al. [108] suggest that ‘personalised micro-competencies’ account for the medical student’s particular contextual considerations, knowledge, skills, experiences, and attitudes, allowing for individualised and contextualised assessment of progress and development. The combination of ‘general micro-competencies’ and ‘personalised micro-competencies’ could be used to alert tutors and host organisations to possible frailties in how the medical student addresses resonance, synchrony, disharmony and or dyssynchrony. This is based on the notion that ‘general micro-competencies’ and ‘personalised micro-competencies’ shape identity work. Appreciation of these gaps then could not only mould the nature of the support but also help with remediation of the issues. This could direct timely and appropriate support that can be directed to medical students, particularly when PIF exhibits a nonlinear [82], longitudinal development [78, 82] process [151]. These insights will also help guide remediation, direct individualized [152] support, counselling [61, 75, 103] and guided reflections [61, 75, 153] in and beyond present postings.

Whilst the use of Tan et al.’s notion of general and personalised ‘micro-competencies’ [148] to benchmark progress underscores the intertwined nature of developing clinical competencies and PIF, program organisers should be mindful of distinguishing the goals and roles of these assessments in supporting PIF longitudinally from assessments of skills, knowledge and attitudes [149, 150].

## Limitations

One of the main limitations of this review has been its overly focused approach that excluded residents and junior doctors in training. This is especially concerning given the longitudinal nature of PIF and the limitations to the number of articles included.

In addition, the nature of PIF also makes comparisons of PIF assessments across different cultures, settings, undergraduate and postgraduate medical student populations. This can be problematic given the differences in levels of experience, competencies, roles, responsibilities and needs and the educational and healthcare programs involved.

Moreover, whilst this study was intended to analyse the wide range of current literature on PIF assessment programs, our review was limited by our specific focus and a lack of consistent reporting of current programs. Furthermore, most of the included papers were largely drawn from North American and European practices, potentially limiting the applicability of these findings in other healthcare settings.

Other limitations include our focus on articles that were published in English which may have compounded concerns over the applicability of these findings beyond the North American and European settings. Whilst taking into account the limited resources and availability of the research and expert teams and limiting the review to the specified dates to increase the chances of completing the review, this too could have seen important articles excluded.

## Conclusion

In mapping current thinking on PIF using the Krishna-Pisupati model, positing the use of general and personalised ‘micro-competencies’ and e-portfolios and proposing the design and employ of a RToP-based tool, this SSR in SEBA proffers unique insights into PIF in Oncology and Palliative Care postings. In doing so, it highlights new areas for study and a chance for stock taking. Even with the possibility of these findings being employed in other postings such as Accident and Emergency and Surgical postings and postgraduate Oncology and Palliative Care training, program designers, administrators and faculty must ensure that their programs do possess the structure, support and means of assessing medical students facing intense clinical, ethical and moral issues in a timely and personalised manner.

Concurrently, further study into the experiences of medical students during their Oncology and Palliative Care postings, the RToP-based tool, the employ of general and personalised ‘micro-competencies’ in tandem with e-portfolios will be the focus of our coming efforts as we look forward to further engagement in this exciting aspect of education in Oncology and Palliative Care.

## Supplementary Information

**Additional file 1: Appendix A.** Full Search Strategy.

**Additional file 2: Appendix B.** Tabulated summaries.

**Additional file 3: Appendix C.** Stage-based nature of PIF**.**

## Data Availability

All data generated or analysed during this review are included in this published article and its supplementary files.
