# Correction: Assessing professional identity formation (PIF) amongst medical students in Oncology and Palliative Medicine postings: a SEBA guided scoping review

**DOI:** 10.1186/s12904-022-01120-1

**Published:** 2023-01-26

**Authors:** Kelly Jia Hui Teo, Mac Yu Kai Teo, Anushka Pisupati, Rui Song Ryan Ong, Chloe Keyi Goh, Claire Hui Xian Seah, You Ru Toh, Neha Burla, Natalie Song Yi Koh, Kuang Teck Tay, Yun Ting Ong, Min Chiam, Warren Fong, Limin Wijaya, Suzanne Pei Lin Goh, Lalit Kumar Radha Krishna

**Affiliations:** 1 Yong Loo Lin School of Medicine, National University of Singapore, 1E Kent Ridge Road, NUHS Tower Block, Level 11, Singapore, 119228 Singapore; 2 Division of Supportive and Palliative Care, National Cancer Centre Singapore, 11 Hospital Crescent, Singapore, 16961 Singapore; 3 Division of Cancer Education, National Cancer Centre Singapore, 11 Hospital Crescent, Singapore, 169610 Singapore; 4 Duke-NUS Medical School, 8 College Road, Singapore, 169857 Singapore; 5 Department of Rheumatology and Immunology, Singapore General Hospital, 16 College Road, Block 6 Level 9, Singapore General Hospital, Singapore, 169854 Singapore; 6 Division of Infectious Disease, Singapore General Hospital, 16 College Road, Block 6 Level 7, Singapore General Hospital, Singapore, 169854 Singapore; 7 KK Women’s and Children Hospital, 100 Bukit Timah Rd, Singapore, 229899 Singapore; 8 Palliative Care Institute Liverpool, Academic Palliative & End of Life Care Centre, University of Liverpool, 200 London Road, Liverpool, L3 9TA United Kingdom; 9 Centre for Biomedical Ethics, National University of Singapore, Blk MD11, 10 Medical Drive, #02-03, Singapore, 117597 Singapore; 10 PalC, The Palliative Care Centre for Excellence in Research and Education, PalC c/o Dover Park Hospice, 10 Jalan Tan Tock Seng, Singapore, 308436 Singapore; 11 Health Data Science, University of Liverpool, Whelan Building, The Quadrangle, Brownlow Hill, Liverpool, L69 3GB UK

**Correction: BMC Palliat Care 21, 200 (2022)**

**https://doi.org/10.1186/s12904-022-01090-4**

Following the publication of the original article [1], the authors became aware that there has been a transposition of the references as changes were made to the tables. This meant that the citations produced and the references in the published manuscript including those displayed in Tables Two to Seven (Table 2, Table, 3, Table 4, Table 5, Table 6, Table 7) does not match. We subsequently corrected this in the manuscript to reflect the correct citations to the references.

The correct referencing for Table 2 is as follows:

**Table:** 

Theories and Framework	Purpose/ Description
Kegan’s constructive development theory [60]	Kegan outlines 6 stages in cognitive development which affects all emotional and relational functioning: Stage 0 (incorporative balance): “in which reflexes are primary”, Stage 1 (impulsive balance): “in which knowing is only about one’s own immediate impulses”, Stage 2 (imperial balance): “in which the individual is aware of concrete and durable categories, that is, her or his own experiences as well as others’ experiences”, Stage 3 (interpersonal balance): “in which abstractions and more mutual relationships become possible”, Stage 4 (institutional balance): “in which understanding of systems, greater autonomy, and self- authorship become possible”, and Stage 5 (interindividual balance): “in which people become the directors and creators of systems, understanding how systems fit together meaningfully”.
Pratt’s theory on professional identity formation [61]	This theory presents PIF as a process of interlinked work and identity cycles, in which identity construction is triggered by work-identity integrity violations which are resolved through 3 identity customization processes – enriching, patching and splinting. A work-identity integrity violation occurs when there is a mismatch between what the individual is doing and what they believe they should be doing in keeping with their professional identity. In individuals with low job discretion and well-developed identities, minor integrity violations are likely to result in identity enrichment. On the other hand, in individuals with low job discretion and major identity violations, patching and splinting occur. In “patching”, an ‘ideal’ identity is adopted used to fill in the deficiencies in the individual’s professional identity, whereas in “splinting”, a former identity is used to protect the currently fragile professional identity.
Wald’s theory on professional identity formation [62]	PIF is “an active, developmental process which is dynamic and constructive and is an essential complement to competency-based education”. PIF “encompasses development of professional values, moral principles, actions, aspirations, and ongoing self-reflection on the identity of the individual and is described ultimately as a complex structure that an individual uses to link motivations and competencies to a chosen career role.” PIF involves “deepening of one’s commitment to the values and dispositions of the profession into habits of mind and heart” and is fundamentally ethical (including an ethic of caring) with development of a set of internal standards or an “internal compass” regulating professionals’ work. Key drivers of PIF “include experiential and reflective processes, guided reflection, formative feedback, use of personal narratives, integral role of relationships and role models, and candid discussion within a safe community of learners (an “authentic community”)”. Three central themes support and reciprocally enhance PIF • Reflective practice: self assessment of values, attitudes, beliefs, reactions to experiences and learning and experiential learning nurture PIF • Relationships: dependent on context and a collaborative environment, mentorship and role modelling, small group collaborative reflection and feedback, peer mentoring, interprofessional work, meaning making and group negotiation • Resilience: responding to stress in a healthy way with ‘bouncing back’ after challenges and growing stronger
Holden’s longitudinal framework through TIME [63]	This framework characterizes the physician identity according to 6 domains which further branch into 30 subdomains. The 6 domains are: attitudes, personal characteristics, duties and responsibilities, habits, relationships, perception and recognition. The framework is mapped onto the 3 developmental phases of medical education (the undergraduate student, the clerkship-level medical student, and the graduating medical student), providing strategies for the longitudinal assessment and promotion of each subdomain at each phase [63].
Korthagen’s level of change model [64]	• The onion model describes 6 different levels on which reflection takes place, including environment, behavior, competencies, beliefs, identity, and, at the model’s center, mission. • Core reflection ◦ Reflection on the level of mission is “concerned with what inspires us, and what gives meaning and significance to our work or our lives”. This is a transpersonal level, involving becoming aware of the meaning of our existence in the world and the role we see ourselves in. ◦ Reflection on the level of identity, on the other hand, is about “how we experience ourselves and our self-concept”. • The inner levels determines how an individual functions on the outer levels and vice versa. • The model shows that aside from behaviour or competencies, there are also other essential qualities of a good teacher.
Barnhoorn’s multi-level professionalism framework [52]	Adapted from Korthagen’s level of change model, this framework delineates 6 levels of influences at which the remediation of unprofessional behavior and development of professional identity can occur. These levels are: environment, behaviour, competencies, beliefs and values, identity, mission. • Environment: “the diverse contexts in which the medical student lives, works and learns, and which influence his or her behavior” • Behaviour: the student’s performance which can be directly observed and assessed • Competencies: the integrated body of knowledge and skills that allows for professional behaviour • Beliefs and values: “the conceptions and convictions a medical student holds true regarding the medical profession and his or her place in it” • Identity: “the way one defines oneself in terms of characteristics, values, and norms, including the characteristics, values, and norms of the profession” • Mission: “the role the medical student sees for him- or her-self in relation to others”
Goldie’s social psychological levels of analysis [16]	Goldie’s social psychological levels of analysis builds on the Personality and Social Structure Perspective (PSSP) model, involving the application of identity formation and identity maintenance process. It classifies medical student’s identity at 3 different levels: ego identity, personal identity, social identity, looking at the interplay between these levels. • Ego identity: “the more fundamental subjective sense of continuity characteristic of the personality” • Personal identity: “at this level students find a fit between their social identity as ‘medical student’ and the uniqueness and idiosyncrasies of their learning/life history” • Social identity: “at this level, the student is most influenced by the pressure to fit into the available identity ‘moulds’ created by cultural and role-related pressures”
Jarvis-Sellinger’s conceptual framework of professional identity formation [65]	A model of action, based on grounded theory, that illustrates how the interactions between context, focus and catalyst aid medical students in processing their emerging professional identities. Within this framework, context refers to the “details medical students use to describe an encounter or activity that has provoked reflection”; focus refers to what the medical students pay attention to in the encounter or activity; catalyst refers to a stimulus such as a learning event that triggers conscious thinking about professional identity within a specific context; while the process “signifies the ways in which medical students experience and describe navigating or negotiating their own emerging professional identities”. Students’ reflections were noted to be focused on either their current identity (being) or their future identity (becoming).
Cruess et al’s schematic representations of professional identity formation and socialization [51]	Within Cruess et al’s schematic representation of professional identity formation, individuals enter medical school with their own identities and through a process of socialization, emerge with both personal and professional identities. The process of socialization involves individuals moving from legitimate peripheral participation in a community to full participation, primarily through social interaction. Socialization is influenced by multiple factors including the healthcare system; learning environment; role models and mentors; clinical and non-clinical experiences; self-assessment; formal teaching and assessment; symbols and rituals; family and friends; attitudes of patients, peers, health care professionals and the public; and isolation from peers.
Hilton and Slotnick’s theory of “proto-professionalism” [66]	The authors propose a broad view of professionalism involving 6 domains, which include areas focusing on doctors alone (ethical practice, reflection and responsibility), and areas requiring collaboration (respect for patients, teamwork and social responsibility). The authors also coin the term “proto-professionalism” to describe the period of learning, experience and maturation to attain professionalism.
Krishna et al’s Ring Theory of Personhood [53]	• Assumes that PIF is part of an individual’s self-concept of personal identity • Suggests that identity can be captured by understanding conceptions of personhood • Identity creates values, beliefs and principles that determine thinking, decision making, conduct and action • The values, beliefs and principles must adapt to new settings, circumstances and these changes result in evolution in identity • Based on the Ring Theory of Personhood that there are 4 elements of identity corresponding to the 4 domains of personhood • The Innate identity draws on Innate Personhood. The Innate Ring is anchored in the belief that all humans are deserving of personhood, “irrespective of clinical status, culture, creed, gender, sexual orientation, religion, or appearance”. The Innate Ring contains gender, name, family identity, religious and cultural, community and nationality-based beliefs, moral values, ethical principles, familial mores, cultural norms, attitudes, thoughts, decisional preferences, roles, and responsibilities (henceforth beliefs, values and principles). These religious, cultural and societal inspired beliefs, values and principles can come into conflict with professional principles and values particularly when contending with withholding and withdrawing treatment [67], care determinations [68], collusion [69], and end-of-life care [70] often tread on Confucian-inspired beliefs [71]. • The Individual Ring contains the unique characteristics and conscious function of the individual [72]. The identity associated with the Individual Ring is informed by the individual’s preferences, biases, beliefs, mores, norms, values and principles and the beliefs, values, and principles of the other rings. Balancing these sometimes-competing considerations in the face of a variety of psychoemotional, experiential, perceptual, and contextual considerations; individual preferences and decision-making styles and biases; and prevailing professional, sociocultural, legal, ethical, and personal considerations can result in dissonance between the different aspect of a medical student’s identity. • The Relational Ring consists of relationships the individual holds to be important. These may come into conflict with legal, ethical, institutional, professional and societal values, beliefs considerations contained in the Societal Ring [73-76]. • When the beliefs, values and principles being instilled are in conflict with those in one of the rings (disharmony) or between the rings of the RToP (dyssynchrony).

The correct referencing for Table 3 is as follows:

**Table:** 

Ethical Framework		Number of articles employing it and references in brackets
Korthagen’s level of change model		One [52]
Barnhoorn’s multi-level professionalism framework		One [79],
Goldie’s social psychological levels of analysis		Eight [51, 52, 62, 78, 80-83]
Kegan’s constructive development theory		Four [63, 78, 81, 82]
Pratt’s theory on professional identity formation		Three [16, 62, 63]
Wald’s theory on professional identity formation		Five [65, 82, 84-86]
Cruess et al’s schematic representations of professional identity formation and socialization		Nine [17, 52, 62, 84, 87-91]
Krishna’s Ring Theory of Personhood		Three [13, 25, 27]
Principles	Information considered (in the context of theories)	Methods of assessment
Longitudinal assessments [5, 28, 63, 78, 79, 85, 88, 90, 92-103]	Personal [13, 17, 25, 27, 51, 52, 60, 65, 66, 78, 79, 81, 82, 88, 89, 91], practical [13, 66, 79, 88], clinical [25, 27, 51, 60, 65, 82, 83, 87-89, 91], environmental [13, 16, 25, 27, 51, 60, 63, 65, 78, 79, 82, 83, 87], academic [25, 82], research [25, 61], systems-based [13, 60, 82, 87, 88, 90];	summative assessments [104]
Multidimensional approach [87, 97, 104, 105]	the medical student’s social [27, 60, 65, 66, 80], personal [13, 16, 17, 25, 27, 51-53, 63, 66, 82, 87, 89], demographic, contextual, academic, research, clinical, and professional values [17, 62, 63, 65, 66, 78-82, 84, 87-91], their **beliefs** [13, 16, 25, 27, 51-53, 60-62, 65, 78, 79, 81-83], **principles** [27, 51, 60, 62, 65, 81, 87, 89], **experiences** [13, 16, 25, 27, 51, 53, 60-63, 65, 66, 79, 81-83, 89-91], **competencies** [17, 51, 52, 62, 63, 66, 80, 82, 84, 87-89], and **goals** [13, 17, 25, 27, 51, 53, 82, 83, 89, 90]	formative assessments [63, 87, 88, 96]
Multimodal approach to assessing PIF [5, 17, 28, 63, 87, 91, 95, 96, 100, 104, 106-111]	environmental conditions, the requirements [87], and influences [62, 66, 78, 89] within the practice setting	use of mixed methods [17, 28, 79, 81, 100, 103, 106, 107, 112-114].
Site-specific assessments [107, 113, 115]	the impact of the formal [16, 51, 52, 65, 78, 79, 82, 87], informal [16, 65, 78, 79, 82, 83, 87], and hidden curriculum [16, 52, 65, 66, 78, 79, 81, 82, 87]	
Assessments at multiple time points [78, 81, 85, 92, 107, 113, 114]	the program and practice expectations [87, 88] on conduct, competencies, attitudes, and goal [13, 51]	
Use of multiple assessors [28, 82, 85, 87, 100, 104, 106, 107, 110, 111, 113, 114, 116, 117]	the medical student’s ethical position [63, 82, 100, 109, 112, 113, 116, 118-121]	
	The medical student’s moral position [81, 84, 87, 95, 100, 112, 118, 119]	
	The medical student’s professional position [52, 63, 81, 82, 90, 91, 98-102, 106, 108, 109, 111-113, 122]	
	The medical student’s values, beliefs and principles - If specific to the medical student: [16, 17, 25, 27, 51-53, 62, 63, 65, 66, 78-82, 84, 87, 88, 90, 91] - If not: [13, 60, 61]	
	The medical student’s actions, attitudes [63], conduct, reflective practice [63] and support mechanisms [63] over time	
	the demographical [84], historical [78], experiential [63, 90] and environmental factors [16, 25, 51, 52, 63, 65, 78, 82, 90] influencing concepts of identity	

The correct referencing for Table 4 is as follows:

**Table:** 

Tools of assessment	
- Guided feedback [101, 122] - Questionnaires [116] - Structured activity - “A Learning Experience” [116] - Brown’s Guide/ BEGAN tool (the Brown Educational Guide to the Analysis of Narrative) [101] - Reflection Evaluation for Enhanced Competences Tool Rubric (REFLECT) [86, 92, 95, 123] - Thematic scoring (“to map and grade the reflection themes”) [95] - Self-reflection and insight scale (SIRS) [115] - Groningen Reflection Ability Scale (GRAS) [115, 124] - Reflective ability rubric [112] - Reflection-in-Learning Scale [116] - Self-assessment [87] - Professional self-identity questionnaire (PSIQ) [105] - Moral reasoning assessment [87] - Observations during clinical assessments [110] - assessment of learning environments [110] - Mentor facilitated conversations [110] - Professional identity essay [81, 119, 120] - Stage-specific attribute scales (SASs) [91] - Physician professional identity survey [97] - Identity integration (IdIn) survey [97] - Developing Scale [84, 91] - Professional identity questionnaire (PIQ) [125]	

The correct referencing for Table 5 is as follows:

**Table:** 

Portfolio contents	
- Material relevant to the roles of the healer and professional [99] - Autobiography [100] - Individual Hippocratic oath document [100] - Health contract [100] - Myers-Briggs personality inventory [100] - Self-grading professional development [127] - Peer feedback [127] - Reflective writing [98, 100, 121, 127] - Essays on physician-patient Relationship [127] - Comment cards [127] - Volunteering experiences [63, 127] - Elective materials [127] - Documentation of other assessments from faculty and peers [127] - Evaluations of standardized patient interactions [127]	

The correct referencing for Table 6 is as follows:

**Table:** 

	Number of articles [88]	References
Beliefs	3	[91, 99, 119]
Mission	0	NA
Abilities/ experiences	23	[80-83, 85, 94, 95, 99-101, 103, 105, 111, 112, 114, 116, 117, 120, 122, 123, 126, 128, 131]
Behaviour	22	[78, 80, 81, 83, 91, 92, 94, 98-100, 102, 106, 108, 109, 111, 114-116, 126, 128, 131, 132]
“Knows”/ “Knows how”	10	[17, 52, 63, 95, 104, 113, 114, 120, 121, 127]
“Shows how”/ “Does”	34	[17, 28, 63, 79, 80, 84, 87, 88, 92-95, 99, 100, 102-104, 106-109, 111, 114, 117, 120, 121, 126-129, 131-134]
“Is”	18	[5, 28, 63, 81, 84, 87, 94, 99, 104, 107, 109, 114, 120, 121, 130, 134]
Reflections	11	[63, 86, 92, 95, 101, 112, 115, 116, 122-124]
Guided feedback	8	[63, 81, 100, 102, 109, 111, 122, 126]
Longitudinal assessments/ portfolio	11	[28, 63, 98-100, 104, 110, 116, 121, 127, 128]
Host organization	4	[88, 103, 126, 131]
Socialisation	13	[63, 81, 82, 86, 90, 98, 100, 101, 104, 112, 115, 116, 127]
Community of Practice	6	[63, 88, 100, 103, 111, 113]
Remediation	11	[79, 87, 89, 103, 107, 115, 118, 127, 131, 133, 135]
Longitudinal assessments	20	[5, 28, 63, 78, 79, 85, 88, 90, 92-103]
Holistic/ multidimensional assessments	4	[87, 97, 104, 105]
Multimodal assessments	16	[5, 17, 28, 63, 87, 91, 95, 96, 100, 104, 106-111].
Site-specific assessments	3	[107, 113, 115]
Multiple timepoints	7	[78, 81, 85, 92, 107, 113, 114]
Multiple assessors	14	[28, 82, 85, 87, 100, 104, 106, 107, 110, 111, 113, 114, 116, 117]

The correct referencing for Table 7 is as follows:

**Table:** 

Tools of assessment	
- Self-assessment [87] - Moral reasoning assessment [87] - Observations during clinical assessments [110] - assessment of learning environments [110] - Mentor facilitated conversations [110] - Professional identity essay [81, 119, 120] - Stage-specific attribute scales (SASs] [91] - Physician professional identity survey [97] - Identity integration (IdIn) survey [97] - Developing Scale [84, 91] - Professional identity questionnaire (PIQ) [125] - Professional self-identity questionnaire (PSIQ) [105]	

The original article has been updated to correct this.

## References

[1] Teo KJH, Teo MYK, Pisupati A, Ong RSR, Goh CK, Seah CHX (2022). Assessing professional identity formation (PIF) amongst medical students in Oncology and Palliative Medicine postings: a SEBA guided scoping review. BMC Palliat Care.

