# Supplementary material for: Assessing professional identity formation (PIF) amongst medical students in Oncology and Palliative Medicine postings: a SEBA guided scoping review
Source: BMC Palliat Care. 2022 Nov 18;21:200. doi: 10.1186/s12904-022-01090-4 (PMC9673314; doi:10.1186/s12904-022-01090-4)
Supplement: Supplementary file 1 — Additional file 1: Appendix A. Full Search Strategy. [file 12904_2022_1090_MOESM1_ESM.docx]

Appendix A. Full Search Strategy

Search Strategies – 1 Jan 2000 – 31 Dec 2021

| **PUBMED** |
| --- |
| ("professional identity formation"[tiab] OR "Professionalism"[Mesh]) AND ("Education, Medical"[Mesh] OR medical[tiab] OR medicine[tiab] OR physician*[tiab] OR doctor*[tiab]) – 1162 |
| **EMBASE** |
| ('professional identity formation'/exp OR ‘professional identity formation’:ti,ab OR ‘identity formation’:ti,ab OR ‘professional identity’:ti,ab OR ‘professional identities’:ti,ab) AND ('medical education'/exp OR 'clinical education'/exp OR ‘physician’/exp OR ‘doctor’/exp) –  1063 |
| **SCOPUS** |
| TITLE-ABS-KEY (professional AND identity AND formation) AND ( TITLE-ABS-KEY ( medicine OR medical OR clinical OR residen* OR physician* ) - 638 |
| **ERIC** |
| “professional identity” AND ("medicine" OR "medical" OR "physician" OR physicians OR "doctor" OR "doctors" OR "resident" OR "residents") – 141 |
