# Supplementary material for: Assessing professional identity formation (PIF) amongst medical students in Oncology and Palliative Medicine postings: a SEBA guided scoping review
Source: BMC Palliat Care. 2022 Nov 18;21:200. doi: 10.1186/s12904-022-01090-4 (PMC9673314; doi:10.1186/s12904-022-01090-4)
Supplement: Supplementary file 2 — Additional file 2: Appendix B. Tabulated summaries. [file 12904_2022_1090_MOESM2_ESM.docx]

Appendix B. Tabulated summaries

| Authors/Year | Article title | Type of study | MERSQI | COREQ | Study Aim | Methodology | Key findings | Proposed solutions/ conclusions |
| --- | --- | --- | --- | --- | --- | --- | --- | --- |
| Aagaard & Moscoso 2019 | **Practical Implications of Compassionate Off- Ramps for Medical Students** | Commentary | NA | NA | “In this issue, Bellini and colleagues describe the scope of this problem and make recommendations to support the implementation of compassionate off-ramps for students.” | NA | NA | “Although this impacts a small proportion of students, the recommendations would help schools achieve the moral imperatives of humanistic care for students while honoring the social contract of the medical profession.” |
| Adams et al. 2019 | **Reflective Writing as a Window on Medical Students’ Professional Identity Development in a**  **Longitudinal Integrated Clerkship** | Qualitative | NA | 117-122 | “This study explores how patient interactions and intentional curriculum design support professional identity construction in students participating in a longitudinal integrated clerkship focused on care for the underserved” | "This qualitative analysis investigated students’ professional identity construction as detailed in 45 reflective essays from 15 students while enrolled in an 11-month longitudinal integrated clerkship (LIC) in a safety-net hospital system. Researchers used an inductive analytic approach." | “Students provided rich and complex descriptions of their experiences. Six themes emerged: care for the underserved; therapeutic alliance; humility and gratitude; altruism; resilience; and aspirations.” | “Professional identity construction was observable through students’ reflective essays. Student interactions with patients provided rich material for professional identity construction, and role models in patient care enhanced this process. This study suggests that clinical learning in a safety-net hospital system, coupled with an LIC curriculum that prioritizes continuity with vulnerable patients, faculty role models, and ongoing reflection, supported the professional identity construction of students as patient-centered caregivers providing equitable care and advocacy for the underserved, described here as an Equity Identity.” |
| Ainsworth & Szauter 2006 | **Medical Student Professionalism: Are We Measuring the Right Behaviors? A Comparison of Professional Lapses by Students and Physicians** | Qualitative | NA | S84-S85 | “Examining the relationship between unprofessional behaviors observed in medical students and those manifested by physicians is important in determining whether medical school faculty are observing and reporting behaviors relevant to medical practice.” | “This study compares the relationship between unprofessional behaviors identified in students at our medical school through Early Concern Notes, and behaviors for which physicians were sanctioned by our state medical board.” | “The majority of reports in both groups were related to lapses in professional responsibility and integrity, and the specific behaviors identified in the groups were similar. A smaller number of reports in both groups were related to pursuit of excellence or personal interactions.” | “There are common features to the professional shortcomings seen in students at our medical school and practicing physicians in our state. These similarities add credibility to our faculty’s observations, and reinforce the relevance of monitoring such behaviors in future physicians.” |
| Al-Eraky & Marei 2016 | **A fresh look at Miller’s pyramid: assessment at the ‘Is’ and ‘Do’ levels** | Perspective | NA | NA | “In its silver jubilee, we celebrate the ground-breaking pyramid of George Miller by submitting a fresh look at it. We discuss two questions. (i) Does the classical pyramidal structure perfectly portray the relationships of the four levels that were described by Miller? (ii) Can the model of Miller fulfill the unmet needs of assessors to measure evolving essential constructs and accommodate the increasingly sophisticated practice of assessment of health professionals?” | NA | “In response to the first question, Miller’s pyramid is revisited in view of two assumptions for pyramidal structures, namely: hierarchy and tapering. Then we suggest different configurations for the same classical four levels and indicate when to use each one. With regard to the second question, we provide a rationale for amending the pyramid with two fur- ther dimensions to assess personal qualities of students at the ‘Is’ level and their performance in teams at the ‘Do’ (together) level.” | “At the end of the article, we yearn to think outside the pyramid and suggest the Assessment Orbits framework to assess students as individuals and in teams.” |
| Ament Giuliani Franco et al. 2020 | **Added value of assessing medical students’ reflective writings in communication skills training: a longitudinal study in four academic centres** | Mixed methods | **10.5** | 1-5 | “This study describes the development and implementation of a model to assess students’ communication skills highlighting the use of reflective writing. We aimed to evaluate the usefulness of the students’ reflections in the assessment of communication skills.” | “Third-year and fourth-year medical students enrolled in an elective course on clinical communication skills development were assessed using different assessment methods.”  “The communication skills course was offered at four universities (three in Brazil and one in Portugal) and included 69 students.  “The students were assessed by a Multiple-Choice Questionnaire (MCQ), an objective structured clinical examination (OSCE) and reflective writing narratives. The Cronbach’s alpha, dimensionality and the person’s correlation were applied to evaluate the reliability of the assessment methods and their correlations. Reflective witting was assessed by applying the Reflection Evaluation for Enhanced Competencies Tool Rubric (Reflect Score (RS)) to measure reflections’ depth, and the Thematic Score (TS) to map and grade reflections’ themes.” | “The Cronbach alpha for the MCQ, OSCE global score, TS and RS were, respectively, 0.697, 0.633, 0.784 and 0.850. The interobserver correlation for the TS and RS were, respectively, 0.907 and 0.816. The assessment of reflection using the TS was significantly correlated with the MCQ (r=0.412; p=0.019), OSCE (0.439; p=0.012) and RS (0.410; p=0.020). The RS did not correlate with the MCQ and OSCE.” | “Assessing reflection through mapping the themes and analysing the depth of reflective writing expands the assessment of communication skills. While the assessment of reflective themes is related to the cognitive and behavioural domains of learning, the reflective depth seems to be a specific competence, not correlated with other assessment methods—possibly a metacognitive domain.” |
| Aukes et al. 2007 | **The development of a scale to measure personal reflection in medical practice and education** | Mixed methods | 10.5 | 178-182 | “Personal reflection is important for acquiring, maintaining and enhancing balanced medical professionalism. A new scale, the Groningen Reflection Ability Scale (GRAS), was developed to measure the personal reflection ability of medical students.” | “Explorative literature study was conducted to gather an initial pool of items. Item selection took place using qualitative and quantitative methods. Medical teachers screened the initial item-pool on relevance, expert-analysis was used for screening the fidelity to the criterion and large samples of medical students and medical teachers were used to investigate the psychometric characteristics of the items. Finally, explorative factor analysis was used to investigate the structure of the scale.” | “The psychometric quality and content validity of the GRAS are satisfactory. The items cover three aspects of personal reflection: self-reflection, empathetic reflection and reflective communication. The 23-item scale proved to be easy to complete and to administer.” | “The GRAS is a practical measurement instrument that yields reliable data that contribute to valid inferences about the personal reflection ability of medical students and doctors, both at individual and group level.” |
| Barnhoorn et al. 2018 | **A practical framework for remediating unprofessional behavior and for developing professionalism competencies and a professional identity** | Perspective | NA | NA | “To this end, the authors propose a multi-level professionalism framework which describes, apart from professional behavior, more levels which influence professional performance: environment, competencies, beliefs, values, identity, and mission.” | NA | NA | “The different levels can provide tools for educators to address and discuss unprofessional behavior with their students in a comprehensive way. By reflecting on all the different levels of the framework, educators guard themselves against narrowing the discussion to either professional behavior or professional identity. The multi-level professionalism framework can help educators and students to gain a better understanding of the root of unprofessional behavior, and of remediation strategies that would be appropriate. For despite the recent emphasis on PIF, unprofessional behavior and its remediation will remain important issues in medical education.” |
| Brainard & Brislen 2007 | **Viewpoint: learning professionalism: a view from the trenches** | Perspective | NA | NA | “We wrote this essay to describe how we are learning medical professionalism as medical students, especially in the hospital environment in our clinical years. We will describe observations and experiences, and relate a few of the many stories collected from students attending a dozen medical schools across the country.” | NA | NA | “The authors maintain that deficiencies in the learning environment, combined  with the subjective nature of professionalism evaluation, can leave students feeling persecuted, unfairly judged, and genuinely and tragically confused. They recommend that administrators, medical educators, residents, and students alike must show a personal commitment to the explicit professionalism curriculum and address the hidden curriculum openly and proactively.” |
| Brokaw et al. 2011 | **Impact of a Competency-Based Curriculum on Medical Student Advancement: A Ten-Year Analysis** | Mixed methods | 10 | 207-211 | “The objective was to document how the Student Promotions Committee (SPC) has adjudicated students’ competency-related deficiencies over the past decade.” | “Using SPC records, the authors determined the frequency of competency-related deficiencies reported to the SPC over time, the nature of those deficiencies, and how the deficiencies were remediated. For the purposes of this study, traditional knowledge-related deficiencies like course failures were excluded from analysis.” | “Using SPC records, the authors determined the frequency of competency-related deficiencies reported to the SPC over time, the nature of those deficiencies, and how the deficiencies were remediated. For the purposes of this study, traditional knowledge-related deficiencies like course failures were excluded from analysis. Results: From 1999 to 2009, 191 students (138 male, 53 female) were referred to the SPC for competency-related deficiencies in 8 performance domains involving communication, basic clinical skills, lifelong learning, self-awareness, social con- text, ethics, problem solving, and professionalism. By comparison, 1,090 students were referred to the SPC for knowledge-related deficiencies during this time. Collectively, the 191 students were cited for 317 separate competency-related deficiencies (M ± SD = 1.7 ± 1.3; range = 1–10). Of these 317 deficiencies, the most prevalent were in the competencies of professionalism (29.3%), basic clinical skills (28.4%), and self-awareness (17.7%). Each of the remaining competencies constituted less than 10% of the total. Successful remediation utilized 12 methods ranging from a simple warning letter to repeating the year under close monitoring. Remediation was unsuccessful for 17 students (8.9%) who were dismissed from medical school primarily due to unprofessional behaviors and poor self-awareness.” | “Competency-related deficiencies can be identified and remediated in most cases, but deficiencies in professionalism and self-awareness are especially challenging.” |
| Buck et al. 2019 | **Challenges to assessing professional identity in medical students: a tale of two measures** | Qualitative analysis | NA | 3-5 | “The purpose of this report is to examine challenges in establishing the validity of measures of identity fusion as one facet of PIF.” | “Utilizing the modern approach of validity as a unitary concept, the authors generated six hypotheses to examine the evidence for the construct validity of the scores of Physician Professional Identity (PPI) and Identity Integration (IdIn), considering relationships of these measures with each other, year of training and data from a larger survey.” | “Responses from 3473 students at 8 medical schools revealed a weak association between the measures with distributions varying by cohort. PPI had a stronger relationship to cohort and IdIn was moderately associated with students’ attitudes relevant to social media use. Responses were independent of response format and evidence supported the interpretation of scores for IdIn as indications of integration of identity” | “Sufficient evidence was found to suggest that these measures assess aspects of PIF. Use of these measures as part of a multidimensional, longitudinal approach to refining understanding of the construct of PIF and developing effective assessment strategies.” |
| Byszewski et al. 2014 | **Socialization to professionalism in medical schools: a Canadian experience** | Qualitative | 8 | 3-8 | “The objective of this study was to compare how professionalism competency is formally addressed in the curricula of Canadian medical schools, and to better understand the Canadian approach to reporting and remediation of lapses.” | “A literature review was performed and with the input of the AFMC(Association of Faculties of Medicine of Canada) Professionalism group, questionnaires were generated. An electronic survey was circulated to key leaders across the country at all the medical schools. In-depth telephone interviews were used to further explore themes, and a subsequent focus group was held to discuss challenges, particularly related to reporting and remediation.” | “The preponderance of formal professionalism teaching remains in the form of lectures and small group sessions in the preclinical years. Formal teaching declines significantly in the clerkship/clinical years. Evaluation is usually performed by a clinical supervisor, but OSCE, portfolio, and concern notes are increasingly used. Role modeling is heavily relied upon in clinical years, suggesting faculty training can help ensure clinical teachers recognize their influence on trainees. Formal remediation strategies are in place at most schools, and often involve essay writing, reflection exercises, or completion of learning modules about professionalism. Lack of clarity on what defines a lapse and fear of reprisal (for both trainees and faculty) limits reporting.” | “This study provides an overview of how professional identity formation is supported in the Canadian context, guided by the standards set out by CanMEDS. Despite a rich literature that describes the definition, program design and evaluation methods for professionalism, in some areas of the curriculum there is still an opportunity to ensure programs embrace the suggested framework. Examples of teaching and evaluation methods, deficiencies in the clinical years of study (clerkship) and challenges in addressing lapses and organizational structure are identified. The results help identify the gaps that need to be addressed and some solutions that can be modeled at other academic institutions.” |
| Chai et al. 2014 | **Feeding: What It Means to Patients and Caregivers and How These Views Influence Singaporean Chinese Caregivers’ Decisions to Continue Feeding at the End of Life** | Quantitative | 6.5 | NA | “To provide a better understanding of the complex interplay of factors that influence the decision by caregivers to continue feeding.” | “Palliative care patients and their main caregiver recruited at National Cancer Centre Singapore between May and July 2011 were included. Data were collected using an interviewer-administered questionnaire.” | “One hundred respondents were included. Three major prevailing themes were identified: filial piety, source of hope, and expression of affection. Nonparametric statistical testing showed that patients and caregivers shared similar views about feeding at the end of life.” | “In this article, we have highlighted the themes of filial piety, hope, and expression of affection that undergird the decision to continue feeding at the end of life. We also found that these views are shared by both patient and caregiver, which are important in helping us understand the carer’s decision-making process when it comes to food. More work needs to be done to further study the concordance between patient and caregiver views with regard to feeding in the palliative care setting.” |
| Chaytor et al. 2012 | **Do students learn to be more conscientious at**  **medical school?** | Quantitative | 10 | NA | “Professionalism in medical students is not only difficult to define but difficult to teach and measure. As negative behaviour in medical students is associated with post-graduate disciplinary action it would be useful to have a model whereby unprofessional behaviour at the undergraduate level can easily be identified to permit appropriate intervention. We have previously developed a scalar measure of conscientiousness, the Conscientiousness Index (CI), which positively correlates to estimates of professional behaviour in undergraduate medical students. By comparing CI points awarded in year 1 and year 2 of study we were able to use the CI model to determine whether teaching and clinical exposure had any effect on students’ conscientiousness.” | “CI points were collected by administrative staff from 3 successive cohorts of students in years 1 and 2 of study. Points were awarded to students for activities such as submission of immunisation status and criminal record checks, submission of summative assignments by a specified date and attendance at compulsory teaching sessions. CI points were then converted to a percentage of maximal possible scores (CI %) to permit direct comparison between years 1 and 2 of study.” | “CI % scores were generally high with each year of study for each cohort showing negatively skewed normal distributions with peaks > 89%. There was a high degree of correlation of CI % scores between year 1 and year 2 of study for each cohort alone and when cohort data was combined. When the change in CI % from year 1 to year 2 for all students was compared there was no significant difference in conscientiousness observed.” | “We have provided evidence that use of a CI model in undergraduate medical students provides a reliable measure of conscientiousness that is easy to implement. Importantly this study shows that measurement of conscientiousness by the CI model in medical students does not change between years 1 and 2 study suggesting that it is a stable characteristic and not modified by teaching and clinical exposure.” |
| Cohen 2006 | **Professionalism in medical education, an American perspective: from evidence to accountability** | Perspective | NA | NA | “the aims of this paper are to provide a definition and framework for professionalism in the context of medical education, describe current threats to medical professionalism, and detail the role medical schools and academic medical centres can play in preparing tomorrow’s doctors to recognise and resist these threats. Additionally, the paper reviews established and potential methods for measuring professionalism and thus assuring public accountability. Finally, specific recommendations are offered for medical schools and teaching hospitals to nurture and sustain professionalism.” | NA | NA | “The progressive intrusion of commercialism into the realm of medicine is threatening to replace the ethics of professionalism with the irreconcilable ethics of the marketplace. Academic medicine must assume greater responsibility and accountability for strengthening the resolve of future doctors to sustain their commitment to the ethics of professionalism. It can do so by improving the medical school admission process, enhancing both formal and experiential teaching of professionalism, and purging the educational environment of unprofessional practices. Ten approaches that aca- demic medicine might adopt to achieve these goals are provided.” |
| Cruess et al. 2014 | **Reframing Medical Education to Support Professional Identity Formation** | Perspective | NA | NA | “The authors propose that a principal goal of medical education be the devel­opment of a professional identity and that educational strategies be devel­ oped to support this new objective.” | NA | NA | “The explicit teaching of professionalism and emphasis on professional behaviors will remain important. However, expanding knowledge of identity formation in medicine and of socializa­ tion in the medical environment should lend greater logic and clarity to the educational activities devoted to ensuring that the medical practitio­ ners of the future will possess and demonstrate the qualities of the “good physician.” |
| Cruess et al. 2015 | **A Schematic Representation of the Professional Identity Formation and Socialization of Medical Students and Residents: A Guide for Medical Educators** | Perspective | NA | NA | “Aim to explicitly support professional identity formation and the ultimate goal of medical education—to ensure that medical students and residents come to “think, act, and feel like a physician.” | NA | NA |  |
| Cruess et al. 2016 | **Talking About Professionalism Through the Lens of Professional Identity** | Perspective | NA | NA | “The recent emphasis on professional identity formation has raised questions about the appropriateness of “Does” as the highest level of aspiration. It is believed that a more reliableindicator of professional behavior is the incorporation of the values and attitudes of the professional into the identity of the aspiring physician. It is therefore proposed that a fifth level  be added at the apex of the pyramid. “ | NA | NA | “As professional identity formation becomes more central to medical education, changes in goals, objectives, and educational strategies are required. Because of the utility of Miller's pyramid, revising it to include an added level – "Is" – appears to be desirable. In this way, the pyramid can continue to serve as a guide to assessment, using adaptations of methods currently in use for assessing professionalism.” |
| Cullen et al. 2016 | **Not all unprofessional behaviors are equal: The creation of a checklist of bad behaviors** | Mixed methods | 12 | 87.9 | “The purpose of this study was to employ policy capturing methodology to develop two empirically validated checklists for identifying professionalism issues in early-career physicians.” | “In a series of workshops, a professionalism competency model containing 74 positive and 70 negative professionalism behaviors was developed and validated. Subsequently, 23 subject matter experts indicated their level of concern if each negative behavior occurred 1, 2, 3, 4, or 5 or more times during a six-month period. These ratings were used to create a “brief” and “extended” professionalism checklist for monitoring physician misconduct.” | “This study confirmed the subjective impression that some unprofessional behaviors are more egregious than others. Fourteen negative behaviors (e.g. displaying obvious signs of substance abuse) were judged to be concerning if they occurred only once, whereas many others (e.g. arriving late for conferences) were judged to be concerning only when they occurred repeatedly.” | “Medical educators can use the professionalism checklists developed in this study to aid in the early identifica- tion and subsequent remediation of unprofessional behavior in medical students and residents.” |
| Daan et al. 2021 | **Validation of the professional identity questionnaire among medical students** | Quantitative | 10 | NA | “Hence in this study, we investigate the validity and reliability of using Brown et al.’s Professional Identity Questionnaire (PIQ) to measure professional identity among medical students.” | “We used the American Psychological Association’s account of validity and reliability to examine the PIQ in terms of its internal structure, its relation to a validated motivation scale, its content, and its internal consistency. To this end, we performed two factor analyses, a Pearson’s correlation test, an expert evaluation and measured Cronbach’s alpha, respectively.” | “Factor analysis revealed two latent factors underlying the items of the PIQ. We found a negative to positive spectrum of Pearson’s correlations corresponding to increasingly internal qualities of motivation. Experts unanimously rated four out of ten of the PIQ’s items as relevant, reliability analysis yielded a Cronbach’s alpha value of 0.82.” | “Despite poor ratings by experts in the field, these results illustrate the PIQ as a valid and reliable quantitative measure of medical students’ professional identity; its two factors reflecting the measure of attached and detached attitudes towards the medical profession. Educators may use the instrument as a tool for monitoring PIF among their students, as well as for designing and evaluating their medical curriculum. Future research might build on the current findings by investigating other dimensions of the PIQ’s validity, including response process validity, predictive validity and consequential validity.” |
| Elliot et al. 2009 | **Shaping professionalism in pre-clinical medical**  **students: Professionalism and the practice of**  **medicine** | Qualitative | NA | 296-301 | “We aimed to develop a novel longitudinal course in professionalism spanning the first 2 years in a medical curriculum.” | “This is a description of the process undertaken over the past 7 years to develop and implement a professionalism curriculum. We used the conceptual framework of constructivism, principles of adult learning, experiential learning and reflective practice to integrate learning with experience. We included student input in session development. Faculty mentors serve as role models to guide, assist and counsel students. Assessment of learners is accomplished using self, peer and mentor evaluation, and a student portfolio. Program evaluation is by course and faculty evaluation.” | “Students are given a final grade of pass or fail, together with a brief narrative. Course evaluations were positive. A survey questionnaire showed that more than 60% of the students reported gaining skills related to course goals.” | “A longitudinal curriculum for the pre-clinical years was successfully launched. Plans are under way to expand this into the clinical years.” |
| Eriksen 2006 | **The Constructive Developmental Theory of Robert Kegan** | Commentary | NA | NA | “This article aims to fill the gap in counselor knowledge by introducing Kegan’s theory and pointing out ways counselors might promote constructive development in clients.” | NA | NA | “Despite these challenges, my experiences indicate that Kegan’s constructive developmental theory offers great richness in understanding people and in understanding how counselors, teachers, and leaders might effectively promote others’ development. Such work is still in its infancy. However, other developmental educators and I have experienced great success in creating developmentally targeted programs and counseling interventions.” |
| Fong et al. 2020 | **Assessment of medical professionalism**  **using the Professionalism Mini Evaluation Exercise (P-MEX) in a multi-ethnic society: a Delphi study** | Mixed methods | 10 | 2-9 | “This study sets out to find consensus on relevance of the items from the Professionalism Mini Evaluation Exercise (PMEX) for assessing medical professionalism in Singapore.” | “A two-round Delphi survey was completed by an expert panel consisting of program directors, associate designated institutional officials, and designated institutional official (n = 15) from residency programs in Singapore. Round 1 comprised of 23 items from the P-MEX rated on a 5-point scale (1 = Definitely include, 2 = Possibly include, 3 = Neutral, 4 = Possibly exclude, 5 = Definitely exclude). In round 2, the experts received feedback from the previous round, and were asked to re-rate the items which did not achieve consensus in the previous round. The threshold for consensus in the study was defined as 70% or greater agreement among experts.” | “Completed questionnaires for both rounds were received from all 15 experts. In round 1, 18 items (78%) achieved consensus to be included. In round 2, 1 (4%) item achieved consensus to be included. However, none of the remaining items achieved consensus to be removed, and they exhibited stability in responses. A list of 19 items covering four domains of medical professionalism (Doctor-patient relationship skills, Reflective skills, Time management and Inter-professional relationship skills) was obtained during the two-rounds of Delphi survey.” | “Nineteen items in the P-MEX had garnered consensus and is suitable for assessing medical professionalism in Singapore. The findings of this study can provide guidance for faculty and institutions who want to assess medical professionalism of their medical trainees.” |
| Goldie 2012 | **The formation of professional identity in medical students: Considerations for educators** | Perspective | NA | NA | "This article conceptualises the processes underlying the formation and maintenance of medical students’ professional identity drawing on concepts from social psychology." | NA | NA | “Identity formation is mainly social and relational in nature. Educators, and the wider medical society, need to utilise and maximise the opportunities that exist in the various relational settings students experience. Education in its broadest sense is about the transformation of the self into new ways of thinking and relating. Helping students form, and successfully integrate their professional selves into their multiple identities, is a fundamental of medical education.” |
| Haruta et al. 2021 | **Validation of the professional self-identity questionnaire for medical students during clinical practice in Japan** | Quantitative | 10.5 | NA | “To validate the Professional Self Identity Questionnaire (PSIQ) for medical students during clinical practice.” | “We conducted a single-year longitudinal question- naire study using the PSIQ. The PSIQ rates the nine items of "teamwork", "communication", "conducting assessment", "cultural awareness", "ethical awareness", "using records", "dealing with emergencies", "reflection", and "teaching" on a scale of 1-7 points. The study participants consisted of 118 fifth- and sixth-grade medical students who completed a mandatory 4-week clinical practice in a community-based medical education (CBME) curriculum. The data were col- lected before and after the CBME curriculum and after clini- cal practice at the time of graduation. To validate the internal structure of the PSIQ, we calculated Cronbach's alpha in the three phases. Additionally, to assess construct validity, we analyzed the trends and differences in each of the nine items of the PSIQ using repeated measures analysis of variance (ANOVA). We also showed the differences in effect size be- fore and after the CBME curriculum.” | “The data of 105 medical students were analyzed. Cronbach's alpha in the three phases was 0.932, 0.936, and 0.939, respectively. PSIQ scores increased progressively for all items, and the F-test for repeated measures ANOVA of nine items' average score across the three phases showed a significant difference F(2,208) = 63.59, p<0.001. The effect size for professional identity of cultural awareness before and af- ter the CBME curriculum was 0.67, or medium.” | “We validated the PSIQ for medical students during clinical practice. Reflecting on professional identity may provide an opportunity for meaningful feedback on readiness to become a doctor.” |
| Hilton & Slotnick 2005 | **Proto-professionalism: how professionalisation occurs across the continuum of medical education** | Perspective | NA | NA | “A concise definition of medical professional- ism is offered that incorporates attributes within 6 domains identified from across the literature on the subject.” | NA | “We suggest 6 domains in which evidence of professionalism can be expected: ethical practice; reflection ⁄ self-awareness; responsibility for actions; respect for patients; teamwork, and social responsibility. Furthermore, we propose that a defining characteristic is encapsulated by the Greek term phronesis, or practical wisdom. Phronesis is acquired only after a prolonged period of experience (and reflection on experience) occurring in concert with the professional’s evolving knowledge and skills base. The prior period we have termed as one of ‘proto-professionalism’. Influences on proto-professionalism are considered in terms of moral and psychosocial development and reflective judgement.” | “Curricula that develop meta-skills will foster the acquisition and maintenance of professionalism. Adverse environmental conditions in the hidden curriculum may have powerful attritional effects.” |
| Ho et al. 2020 | **The impact of death and dying on the personhood of medical students: a systematic scoping review** | Systematic review | NA | NA | “A systematic scoping review (SSR) is proposed to map available data to guide the design of much needed sup- port systems for these medical students.” | “We adopt Krishna’s Systematic Evidence Based Approach to carry out a Systematic Scoping Review (SSR in SEBA) on the impact of death and dying on medical students. This structured search process and concurrent use of thematic and directed content analysis of data from six databases (Split Approach) enhances the transparency and reproducibility of this review.” | “Seven thousand six hundred nineteen were identified, 149 articles reviewed and 52 articles included. The Split Approach revealed similar themes and categories that correspond to the Innate, Individual, Relational and Societal domains in the Ring Theory of Personhood.” | “Facing death and dying amongst their patients affect how medical students envisage their personhood. This underlines the need for timely, holistic and longitudinal support systems to ensure that problems faced are addressed early. To do so, there must be effective training and a structured support mechanism.” |
| Hodges et al. 2019 | **Assessment of professionalism: From where have we come – to where are we going? An update from the Ottawa Consensus Group on the assessment of professionalism** | Systematic review | NA | NA | “There were several objectives identified. One goal was to conduct a bibliometric analysis to document how the original 2011 report had been taken up and for what purposes. Another goal was to examine the degree to which calls for research in the original report had been heeded. A third goal was to identify changes or emerging innovations in practices of assessment.” | “A bibliometric study identified all publications on assessment of professionalism since 2011, noting those that cited the original report. Articles were coded to identify the reason for citation and new trends in assessment. Bibliometric data were supplemented by discussion groups held at key international education meetings.” | “Six-hundred publications on the assessment of professionalism were found in Google Scholar and 164 in Web of Science since 2011, of which 177 (30%) and 84 (50%) respectively cited the original IWG publication. English language publications were most common (83%), but there were articles in 13 other languages by authors from 40 countries. The report was cited commonly to justify attention to professionalism in general (41%), assessment of professionalism (38%) and to explore professionalism in different countries and professions (25%). A thematic analysis showed that of 9 research areas recommended in 2011, 7 of 9 categories were represented with a large increase in research across languages and cultures.” | “Though the assessment of professionalism remains a challenge the research base continues to grow, especially related to professionalism across cultures and languages, and a large percentage of publications cite the IWG recommendations. There remains a gap in research and writing about patients’ perspectives.” |
| Hoffman et al. 2016 | **Is Reflective Ability Associated With**  **Professionalism Lapses During Medical School?** | Quantitative | 12.5 | NA | “This study explored the association between reflective ability and professionalism lapses among medical students.” | “The authors conducted a retrospective case–control study of all students who matriculated at Indiana University School of Medicine from 2001 to 2009. The case group (n = 70) included those students who had been cited for a professionalism lapse during medical school; the students in the control group (n = 230) were randomly selected from the students who had not been cited for a professionalism lapse. Students’ professionalism journal entries were scored using a validated rubric to assess reflective ability. Mean reflection scores were compared across groups using t tests, and logistic regression analysis was used to assess the relationship between reflective ability and profession­alism lapses.” | “Reflection scores for students in the case group (2.46 ± 1.05) were significantly lower than those for students in the control group (2.82 ± 0.83) (P = .01). A lower reflection score was associated with an increased likelihood that the student had been cited for a professionalism lapse (odds ratio = 1.56; P < .01).” | “This study revealed a significant relationship between reflective ability and professionalism, although further study is needed to draw any conclusions regarding causation. These findings provide quantitative evidence to support current anecdotal claims about the relationship between reflection and professionalism.” |
| Holden et al. 2012 | **Professional Identity Formation in Medical Education: The Convergence of Multiple Domains** | Commentary | NA | NA | “In this study, the authors review essential elements of these three domains, identify features relevant to medical PIF, and describe strategies reported in the medical education literature that may influence PIF.” | NA | NA | “Thus the work to date examining PIF in medical students reflects themes from the multiple domains of professionalism, identity development and formation. Guided reflection, internalization, relationship building, the need for role models, the need for early experiences, and the processes of exploration and commitment are themes that appear throughout these interrelated domains. Yet each perspective brings an important and unique lens to the multifaceted phenomenon of identity construction. More detailed explication of the process of PIF in medical education should involve application of the principles and frameworks from these varied disciplines. “ |
| Holden et al. 2015 | **Professional Identity Formation: Creating a Longitudinal Framework Through TIME (Transformation in Medical Education)** | Descriptive | NA | NA | “ In this article, the authors describe the task force’s process for defining PIF and developing a framework, which includes 10 key aspects, 6 domains, and 30 subdomains to characterize the complexity of physician identity. The task force mapped this framework onto three developmental phases of medical education typified by the undergraduate student, the clerkship-level medical student, and the graduating medical student. The task force provided strategies for the promotion and assessment of PIF for each subdomain at each of the three phases, in addition to references and resources.” | NA | NA | “The authors emphasize the importance of longitudinal, formative assessment using a combination of existing assessment methods. Though not unique to the medical profession, PIF is critical to the practice of exemplary medicine and the well-being of patients and physicians. Though not unique to the medical profession, PIF is critical to the practice of exemplary medicine and the well-being of patients and physicians.” |
| Huang et al. 2021 | **Impact of Dying Neonates on Doctors' and Nurses' Personhood: A Systematic Scoping Review** | Systematic review | NA | NA | “In mapping prevailing data on the impact of caring for dying neonates on HCPs, an SSR proffers new insights on changes to their beliefs, values, coping, actions, relationships and self-concepts of personhood.” | “Krishna’s Systematic Evidenced Based Approach (SEBA) supports a robust, reproducible review whilst its constructivist approach and relativist lens effectively contends with diverse data sources, facilitating a holistic study.” | “A total of 9826 abstracts were reviewed and 69 articles were included. Thematic and content analysis were used simultaneously. The four categories drawn from the Ring Theory of Personhood (RToP) complements the four themes identified: Emotional and Physical Difficulties, Coping Mechanisms, Conflict and Recommendations.” | “In highlighting significant longitudinal effects upon all aspects of their lives, this SSR in SEBA reveals a critical need for timely, appropriate and personalized support. We recommend adapting the RToP as an assessment tool to identify and evaluate the needs of these HCPs. It may also be used to structure a holistic support mechanism. Future studies are required to validate its use and appraise other aspects of support available for HCPs.” |
| Humphrey et al. 2007 | **Promoting an Environment of Professionalism: The University of Chicago “Roadmap”** | Descriptive | NA | NA | “In 2005, the University of Chicago Pritzker School of Medicine unveiled an institution-wide Roadmap to Professionalism designed to both increase awareness about issues of medical professionalism across the institution and gain a better understanding of how medical trainees’ professional behaviors’ change during their training as a result of the medical learning environment. The authors describe the institution’s approach and progress to date.” | NA | NA | “This institution’s approach and experience to date may provide valuable lessons for educators and leaders aiming to assess and improve their learning environments.” |
| Irby & Hamstra 2016 | **Parting the Clouds: Three Professionalism Frameworks in Medical Education** | Perspective | NA | NA | “In this article, the assumptions and contributions of each framework are described to provide greater insight into the nature of professionalism. By examining each discourse in detail, underlying commonalities and differences can be highlighted to assist educators in more effectively creating professionalism curricula, pedagogy, and assessment.” | “We examine the assumptions made by each framework and highlight both the historical and theoretical contexts that led to the crystallization of these assumptions” | “Our analysis suggests that no one framework is adequate for describing professionalism and that, in considering the features of each framework in turn, or as part of a whole, we gain a deeper understanding of the term.” | “From our perspective, current conflicts in medical education over curriculum, pedagogy, and assessment of professionalism are rooted in the differing assumptions underlying each framework. By better understanding them, we hope that the conversations and dialogue around professionalism can be clarified and made more transparent to teachers and learners alike.” |
| Iserson 2018 | **Talking About Professionalism Through the Lens of Professional Identity** | Commentary | NA | NA | “This paper presents a method for educators to help recipients understand how adhering to professionalism’s basic elements will affect their career trajectory.” | NA | NA | “Those tasked with discussing professionalism with physicians, other health care professionals, and trainees may want to consider approaching the topic tangentially, through the lens of professional identity.” |
| Jarvis-sellinger et al. 201 | **Understanding Professional Identity Formation in Early Clerkship: A Novel Framework** | Qualitative | NA | 1574-1579 | “This study examined third-year medical students’ experiences of professional identity formation (PIF) during clinical clerkship.” | “The authors relied on an interpretivist perspective, informed by a grounded theory approach, to analyze data, which were collected from a pilot course designed to support medical students’ efforts to “unhide” the hidden curriculum in relation to their development as medical students and emerging professionals.” | “Twelve third-year medical students engaged in 10 collaborative discussions with 3 faculty members, a resident, and a fourth-year student (2015–2016). Discussions facilitated students’ reflection on their professional journeys. Analysis of transcribed discussions resulted in a conceptual framework useful for exploring and understanding students’ reflections on their PIF. Through analyzing students’ experiences, the authors identified 4 components that constituted PIF stories: context, focus, catalyst, process.” | “The analysis resulted in the development of a conceptual framework and distinct identity formation themes. Discrete reflections focused on either students’ current identity (being) or their sense of future self (becoming). The study identified catalysts that sparked participants’ introspection about, or their processing of, identity. The moments that generate profound feelings of awareness in students are often moments that would not be recognizable (even post hoc) as remarkable by others.” |
| Jarvis-sellinger et al. 2012 | **Competency is Not Enough: Integrating Identity Formation into the Medical Education** | Perspective | NA | NA | “To address these concerns, the authors argue that an expanded approach be taken that includes a focus on professional identity development.” | NA | NA | “Reflection on the relationship between social roles, professional identity, and individual competence specific to a particular community of practice is, therefore, the critical process linking social structures with individual behavior.” |
| Jha et al. 2016 | **A Framework for Understanding Lapses in Professionalism Among Medical Students: Applying the Theory of Planned Behavior to Fitness to Practice Cases** | Descriptive | NA | 1622-1626 | “In this article, the authors propose the use of the theory of planned behavior (TPB) as a framework to help evaluate unprofessional behavior in students. The TPB is a deliberative processing model that explains how  an individual’s behavior is underpinned by his or her cognitions, with behavior being primarily dependent on the intention to perform the behavior (behavioral intention). Intention, in turn, is determined by three variables: attitude, subjective norm, and perceived behavioral control.” | NA | NA | “We propose that the TPB, with its inherent requirement for the incorporation of behavioral intention (via attitude, subjective norm, and perceived behavioral control) into FTP judgments, provides a framework to facilitate this process.” |
| Kalet et al. 2007 | **Promoting Professionalism through an Online Professional Development Portfolio: Successes, Joys, and Frustrations** | Qualitative | NA | 1065-1066 | “The authors developed and implemented a program built around a Web-based Professional Development Portfolio (PDP) to assess and document professional development in medical students at New York University School of Medicine. In this article, the authors describe the development of the PDP and share four years of experience with its implementation. We describe the experiences and attitudes of the first students to participate in this program as reported in an annual student survey.” | “This program requires students to regularly document their professional development through written reflections on curricular activities spanning preclinical and clinical years. Students post reflections, along with other documents that chronicle their professional growth, to their online PDP. Students meet annually with a faculty mentor to review their portfolios, assess their professional development based on predetermined criteria, and establish goals for the coming year.” | “Students’ experiences of and satisfaction with the PDP was varied. The PDP has been a catalyst for honest and lively debate concerning the meaning and behavioral manifestations of professionalism. A Web-based PDP promoted self-regulation on an individual level because it facilitated narrative reflection, self-assessment, and goal setting, and it structured mentorship.” | “Therefore, the PDP may prepare students for the self-regulation of the medical profession—a privilege and obligation under the physician’s social contract with society.” |
| Kalet et al. 2017 | **Measuring professional identity formation early in medical school** | Mixed methods | 8 | 255-259 | "To assess the feasibility and utility of measuring baseline professional identity formation (PIF) in a theory-based professionalism curriculum for early medical students." | “All 132 entering students completed the professional identity essay (PIE) and the defining issues test (DIT2). Students received score reports with individualized narrative feedback and wrote a structured reflection after a large-group session in which the PIF construct was reviewed. Analysis of PIEs resulted in assignment of a full or transitional PIF stage (1–5). The DIT2 score reflects the proportion of the time students used universal ethical principles to justify a response to 6 moral dilemma cases. Students’ reflections were content analyzed.” | “PIF scores were distributed across stage 2/3, stage 3, stage 3/4, and stage 4. No student scores were in stages 1, 2, 4/5, or 5. The mean DIT2 score was 53% (range 9.7–76.5%); the correlation between PIF stage and DIT score was q 1⁄4 0.18 (p 1⁄4 0.03). Students who took an analytic approach to the data and demonstrated both awareness that they are novices and anticipation of continued PIF tended to respond more positively to the feedback.” | “These PIF scores distributed similarly to novice students in other professions. Developmental-theory based PIF and moral reasoning measures are related. Students reflected on these measures in meaningful ways suggesting utility of measuring PIF scores in medical education.” |
| Kalet et al. 2021 | **Does a measure of Medical Professional Identity Formation predict communication skills performance?** | Quantitative | 13.5 | NA | “To validate an approach to measuring professional identity formation (PIF), we explore if the Professional Identity Essay (PIE), a stage score measure of medical professional identity (PI), predicts clinical communication skills.” | “Students completed the PIE during medical school orientation and a 3-case Objective Structured Clinical Exam (OSCE) where standardized patients reliably assessed communication skills in 5 domains. Using mediation analyses, relationships between PIE stage scores and communication skills were explored.” | “For the 351 (89%) consenting students, controlling for individual characteristics, there were in- creases in patient counseling (6.5%, p<0.01), information gathering (4.3%, p=0.01), organization and management (4.1%, p = 0.02), patient assessment (3.6%, p = 0.04), and relationship development (3.5%, p=0.03) skills for every half stage increase in PIE score. The communication skills of lower socio-economic status (SES) students are indirectly impacted by their slightly higher PIE stage scores.” | “Higher PIE stage scores are associated with higher communication skills and lower SES. PIE predicts critical clinical skills and identifies how SES and other characteristics indirectly impact future clinical performance, providing validity evidence for using PIE as a tool in longitudinal formative academic coaching, program and curriculum evaluation, and research.” |
| Khan 2018 | **The effects of teaching professionalism in a private medical college of Pakistan** | Mixed methods | 12 | 1095-10907 | “To assess the response of medical students towards different elements of professionalism, after its formal teaching and to explore the views of students about the effectiveness of various strategies used to teach professionalism and how to improve them.” | “The Penn State College of Medicine Professionalism Questionnaire was used to gather the perceptions of MBBS students about the elements of professionalism after its formal training. Focused group discussions (FGDs) conducted to explore students’ understanding of the effectiveness of various strategies used to teach professionalism were audio recorded, transcribed and analyzed by thematic analysis with the software NVivo. Quantitative data was analyzed by SPSS version 21.” | “There were 300 students. The mean age of students was 20.0 ± 1.55 years. Females were 67%. Eighty eight percent of students had FSc education and 12% had completed A levels. The medical students of all four MBBS classes considered the six attributes of professionalism as important. The Cronbach alpha value for all the elements of professionalism in four classes was above 0.75. Mean scores calculated for the elements of professionalism for the first, second, third and fourth year students was 145.66 (± 21.05), 130.98 (± 24.67), 121.09 (±17.13) and 151.34 (±12.28) respectively. There were significant differences in the mean scores among four classes of MBBS (p=0.000). Role modeling was determined to be the most effective and useful method to inculcate professionalism among medical students. In two focused group discussions six major themes were identified by the students including; professionalism training, role modeling, faculty development, mentoring, student to student counseling, and assessment of professionalism.” | “A robust curriculum with explicit teaching of professionalism does not only uphold and maintain the pre-training values of medical students but also brings about a significant improvement in their attitudes pertaining to professionalism. The students recognize role modelling to be the most effective method in developing professionalism. They perceive that teaching strategies based on role modelling, formal mentoring, faculty development and formal assessment plan can improve the training of professionalism.” |
| Korthagen & Vasalos 2005 | **Levels in reflection: core reflection as a means to enhance professional growth** | Perspective | NA | NA | “In the present article we examine this problem and demonstrate that often a form of reflection is needed which does go deeper, and which we call core reflection. This form of reflection, however, does not entail delving into a person’s private life, but it can lead to profound changes. Moreover, it is possible to train educators to reach these more deeply rooted aspects.” | NA | NA | “In sum, we feel that core reflection, like ordinary reflection, is of crucial importance for teacher educators, teachers, and pupils, and may lead to a shift in our thinking about (teacher) education, a shift concurring with the shift towards positive psychology.” |
| Krishna 2013 | **Palliative care imperative: A framework for holistic and inclusive palliative care** | Perspective | NA | NA | “To meet the need for a more flexible and inclusive ethical framework, this paper attempts to meld the prevailing expectations of a deontic-inspired Duty of Palliative Care (DoPC) at the end-of-life with an aretaic-inspired Virtue Ethics framework to better capture the nuances of end-of-life decision making in Singapore.” | NA | NA | “The PCI addresses the shortcomings of a universal, rational, and impassive deontic framework that chooses to neglect the nuances of the individual. In addition, it does not succumb to the allure of an overly individualistic moral framework; fighting this allure by utilising the specific duties set up within the DoPC to reign in expectations.” |
| Krishna et al. 2014 | **Limits to relational autonomy—The Singaporean experience** | Perspective | NA | NA | “Taking into account local data and drawing upon a wider concept of personhood that extends beyond prevailing family-centric ideals along with the complex interests that are focused upon the preservation of the unique nature of personhood that arises from the Ring Theory of Personhood, we propose and ‘‘operationalize’’ the employing of an authoritative welfare-based approach, within the confines of best interest decision making, to better meet the current care needs within Singapore.” | NA | NA | “Our model does not prevent free choice but merely ensures that any proposed action be within the remit of acceptable practice and the patient’s best interests. We hope that such practice will protect the interests and choices of patients and be readily imbibed within similar practice settings particularly, given its consonance with Confucian-inspired ancient Chinese Medical Ethics.” |
| Krishna 2015 | **Accounting for personhood in palliative sedation: the Ring Theory of Personhood** | Perspective | NA | NA | “Here I proffer a clinically based alternative to this view, the Ring Theory of Personhood, which dispels these concerns about sedation at the end of life.” | NA | NA | “This ethically sensitive, clinically relevant, culturally appropriate concept of personhood instead sees personhood in a wider, flexible, more patient-centred manner than prevailing concepts of personhood and better reflects the nuanced views of patients themselves as they attempt to preserve their personhood in increasingly difficult circumstances.” |
| Krishna 2015 | **Addressing the Concerns Surrounding Continuous Deep Sedation in Singapore and Southeast Asia: A Palliative Care Approach** | Perspective | NA | NA | “I argue that reinstituting a palliative care-inspired approach that includes a holistic review of the patient’s situation and the engagement of a multidisciplinary team (MDT) guided by clearly defined practice requirements that have been lacking amongst many prevailing guidelines will overcome prevailing objections to this practice and allow for the legitimization of this process.” | NA | NA | “CDS should be seen as an appropriate, valid, and effective means of treating intractable suffering at the end of life when the key prerequisites for its application are met.” |
| Krishna & Alsuwaigh 2015 | **Understanding the fluid nature of personhood – The Ring Theory of Personhood** | Descriptive | NA | 172-181 | “From the data gathered, a culturally appropriate, clinically relevant and ethically sensitive concept of personhood was proposed: the Ring Theory of Personhood (Ring Theory) that better captures the nuances of local conceptions of personhood.” | NA | NA | “We believe that our findings are reflective of the Singaporean oncology patient population given that participants closely mirrored local racial and religious demographics. In order to build upon our findings we propose undertaking further studies with a larger linguistic pool and sample size.” |
| Krishna & Ho 2015 | **Reapplying the 'Argument of Preferable Alternative' Within the Context of Physician-Assisted Suicide and Palliative Sedation.** | Perspective | NA | NA | “By examining the concepts and terminology in PS from both individual and collective views within different guidelines and the assumptions of APA, we suggest that PS is too variable in practice to harness the APA.” | NA | NA | “From both an individual and collective view, the concepts of PS remain too unstable to harness the “argument of preferable alternative”. At present, one should instead wait for a more focused and well-demarcated concept that can be applied in a more consistent manner replete for PS to even warrant con- sideration as a viable alternative to PAS.” |
| Krishna et al. 2015 | **Advancing a Welfare-Based Model in Medical Decision** | Perspective | NA | NA | “In this paper we will highlight the inherent flaws in the proposed Relational Autonomy (RA) framework as it attempts to bridge the differences between principlism and regnant familialism, and forward a flexible patient-centred, context-sensitive, welfare-based model of care that we believe will better address” | NA | NA | “We continue to work toward validating our welfare-based approach within the clinical setting and look forward to proffering an ethically sensitive, clinically relevant, culturally sensitive approach applicable to the various clinical scenarios faced by clinicians today.” |
| Krishna et al. 2015 | **Applying the Welfare Model to At-own-risk discharges** | Perspective | NA | NA | “With the validity of at- own-risk discharge request in question and the welfare and patient interest at stake, an alternative approach to assessing at-own-risk discharge requests are called for. The welfare model circumnavigates these concerns and preserves the patient’s welfare through the employ of a multidisciplinary team guided holistic appraisal of the patient’s specific situation that is informed by clinical and institutional standards and evidenced-based practice. The welfare model provides a robust decision-making framework for assessing the validity of at-own-risk discharge requests on a case-by-case basis.” | NA | NA | “For the present time, the WM represents the best means of ensuring a balanced, accountable, transparent means to protect patient welfare in the local and indeed many other family-centric settings.” |
| Kuek et al. 2020 | **The impact of caring for dying patients in intensive care units on a physician's personhood: a systematic scoping review** | Systematic review | NA | NA | “Supporting physicians in Intensive Care Units (ICU)s as they face dying patients at unprecedented levels due to the COVID-19 pandemic is critical. Amidst a dearth of such data and guided by evidence that nurses in ICUs experience personal, professional and existential issues in similar conditions, a systematic scoping review (SSR) is proposed to evaluate prevailing accounts of physicians facing dying patients in ICUs through the lens of Personhood. Such data would enhance understanding and guide the provision of better support for ICU physicians.” | “An SSR adopts the Systematic Evidenced Based Approach (SEBA) to map prevailing accounts of caring for dying patients in ICUs. To enhance the transparency and reproducibility of this process, concurrent and independent use of tabulated summaries, thematic analysis and directed content analysis (Split Approach) is adopted.” | “Eight thousand three hundred fifty-eight abstracts were reviewed from four databases, 474 full-text articles were evaluated, 58 articles were included, and the Split Approach revealed six categories/themes centered around the Innate, Individual, Relational and Societal Rings of Personhood, conflicts in providing end of life care and coping mechanisms employed.” | “This SSR suggests that caring for dying patients in ICU impacts how physicians view their personhood. To resolve conflicts within individual concepts of personhood, physicians use prioritization, reframing and rely on accessible, personalized support from colleagues to steer coping strategies. An adapted form of the Ring Theory of Personhood is proposed to direct timely personalized, appropriate and holistic support.” |
| Loh et al. 2015 | **Place of Care at End of Life: What Factors Are Associated With Patients’ and Their Family Members’ Preferences?** | Quantitative | 8 | NA | “This pilot study describes the perceptions of Singaporean patients with cancer and their family members that affect their choices in place of care.” | “Patients with cancer and their family members were surveyed at a tertiary hospital in Singapore. This convenience sample was recruited from April to July 2012.” | “Fourteen pairs of patients and their family members (N 1⁄4 28) were recruited. A majority of patients (64.3%) and family members (71.4%) were found to have a preferred place of care at the end of life. Of the respondents who expressed a preference (n 1⁄4 19), 88.9% of patients and 90.0% of family members named ‘‘home’’ as their preferred place of care. Quality of care at home was rated ‘‘good’’ or ‘‘excellent’’ by all patients and 85.7% of family members.” | “Home is the most favored choice among patients and family members who have a preferred place of care.” |
| Macneill et al. 2020 | **A professionalism program in medical education and training – From broad values to specific applications: YLL School of Medicine, Singapore** | Descriptive | 8 | NA | “The process for introducing and developing a program for teaching medical professionalism at the  National University of Singapore, School of Medicine is outlined.” | NA | NA | “The results are confirming in that: the majority of recommendations have been implemented; the program has developed further; and is  supported by ancillary student enrichment activities. Medical professionalism has been given prominence through all phases of the course. Nevertheless, challenges remain and particularly in  the extent to which medical professionalism is taught and assessed in various clinical postings.” |
| Mak van-der Vossen et al. 2013 | **How we designed and implemented teaching, training, and assessment of professional behaviour at VUmc School of Medical Sciences Amsterdam** | Descriptive | NA | 710-714 | “In this paper we describe how we designed and implemented teaching, training, and assessment of PB at VUmc School of Medical Sciences Amsterdam.’ | “We designed an educational theme, Professional Behaviour (PB), as a longitudinal thread throughout our six-year curriculum after defining PB as ‘‘The observable aspects of practising professionalism’’. This definition was translated into a set of practical skills that can be observed: ‘‘The ability to deal with tasks, to deal with others and to deal with oneself’’.” | “We assess PB 29 times in the course of the medical curriculum. Students with an unsatisfactory PB do not get their degree irrespective of their medical knowledge. We train teachers to identify and report unprofessional student behaviour, and we offer these students interventions and support.” | “With the educational theme ‘‘Professional Behaviour’’ we have defined PB for our institute and firmly embedded it in the medical curriculum. We use workplace learning and role models for teaching PB. Different teachers carry out multiple formative and summative assessments, using standardized assessment scales. With these measures we intend to promote a culture of excellence in PB in our institute.” |
| Mak van-der Vossen et al. 2016 | **Distinguishing Three Unprofessional Behavior Profiles of Medical Students Using Latent Class Analysis** | Qualitative | 11 | NA | “The authors aimed to identify patterns in the unprofessional behaviors of medical students and to construct descriptions based on these patterns.” | “Content analysis of research articles yielded a template of unprofessional behaviors for coding student evaluation forms indicating unsatisfactory professional behavior, collected from 2012 to 2014 at the VUmc School of Medical Sciences, Amsterdam, the Netherlands. Latent class analysis was used to identify classes of students with a high chance of displaying comparable unprofessional behaviors. Teachers’ feedback of prototype students was summarized to generate profile descriptions.” | “A template of 109 behaviors was used to code 232 evaluation forms of 194 students (3.9% students/ year). Latent class analysis identified three hypothetical classes of students: Class 1 (43%) was labeled as “Poor reliability,” class 2 (20%) was labeled as “Poor reliability and poor insight,” and class 3 (37%) was labeled as “Poor reliability, poor insight, and poor adaptability.” | “These profiles of unprofessional behavior might help to improve the evaluation of unprofessional behavior in medical school. Further research should provide evidence for confidently accepting or rejecting the profiles as an instrument to identify which students are expected to benefit from remediation trajectories.” |
| Mak-van der Vossen et al. 2014 | **Assessing professional behaviour: Overcoming teachers’ reluctance to fail students** | Mixed methods | 10.5 | 2-4 | “Developing professional behaviour is an important goal of medical education in which teachers play a significant part. Many teachers can be reluctant to fail students demonstrating unprofessional behaviour. We hypothesize that supporting teachers in teaching and assessing professional behaviour and involving them in remediation will reduce this reluctance.” | “In 2010, VUmc School of Medical Sciences Amsterdam introduced an educational theme on professional behaviour for the bachelor's and master's programmes in medicine with a special emphasis on supporting teachers in teaching and assessing professional behaviour and involving them in the remediation process. Information was extracted from the student database on the number of unprofessional behaviour judgments awarded over 2008-2010 (before the intervention), and 2010-2013 (after introducing the intervention), which was compared. To find out if teachers' reluctance to fail had decreased, qualitative feedback from the teachers was gathered and analysed” | “Since the implementation of the educational theme, the number of unprofessional behaviour judgments has risen. The teachers are positive about the implemented system of teaching and assessing professional behaviour, and feel less reluctant to award an unsatisfactory professional behaviour judgment.” | “Supporting teachers in teaching and assessing professional behaviour and involving them in students' remediation appears to reduce their reluctance to fail students demonstrating unprofessional behaviour.” |
| Mak-van der Vossen et al. 2020 | **How to identify, address and report students’ unprofessional behaviour in medical school** | Descriptive | NA | NA | “This AMEE guide provides a research overview of the identification of, and responding to unprofessional behaviour in medical students. It is directed towards medical educators in preclinical and clinical undergraduate medical education. It aims to  describe, clarify and categorize different types of unprofessional behaviours, highlighting students’ unprofessional behaviour profiles and what they mean for further guidance.” | NA | NA | “By giving feedback to each other, and talking about unprofessionalism both students and educators can potentially learn. Students can learn that unprofessionalism is not tolerated, since it has a negative effect on (future) patient care. Educators can learn which factors in the educational context need to be influenced to support professional behaviour of medical students” |
| Mann et al. 2007 | **Reflection and reflective practice in health professions education: a systematic review** | Systematic review | NA | NA | “We, therefore, designed a literature review to evaluate the existing evidence about reflection and reflective practice and their utility in health professional education. Our aim was to understand the key variables influencing this educational process, identify gaps in the evidence, and to explore any implications for educational practice and research.” | “The PubMed, CINAHL, and PsychInfo databases were searched using the following keywords: action; experience; insight; journal; personal; portfolio; professional; reflect; reflection; reflective practice; self-aware; and, self manage. We also conducted hand searches and reviewed bibliographies of identified papers. The search was limited to English language papers published between 1995 and 2005, dealing specifically with medical or health professional education or practice.” | “The original search identified more than 600 papers, commentaries and reviews of the literature. To address our specific interest, we excluded all papers that did not describe research examining the process and outcomes of reflective practice in health professional education and practice. This resulted in the identification of 29 papers.” | “In this review, we have synthesized the findings of 29 studies of reflective practice in the health professions. While the literature is early in its development, certain findings were quite consistent across professions and levels of learners. The very nature of reflective practice makes its quantification challenging. Yet, as understanding of reflection develops and the field matures, there will be a need for studies with rigorous designs that will allow us to evaluate the effect of different educational strategies to promote its development.” |
| Matsuyama et al. 2021 | **Limited effects from professional identity formation-oriented intervention on self- regulated learning in a preclinical setting: a randomized-controlled study in Japan** | Quantitative | 14 | NA | “Developing self-regulated learning in preclinical settings is important for future lifelong learning. Previous studies indicate professional identity formation, i.e., formation of self-identity with internalized values and norms of professionalism, might promote self-regulated learning. We designed a professional identity formation- oriented reflection and learning plan format, then tested effectiveness on raising self-regulated learning in a preclinical year curriculum.” | “A randomized controlled crossover trial was conducted using 112 students at Jichi Medical University. In six one-day problem-based learning sessions in a 7-month pre-clinical year curriculum, Groups A (n = 56, female 18, mean age 21.5y ± 0.7) and B (n = 56, female 11, mean age 21.7y ± 1.0) experienced professional identity formation- oriented format: Group A had three sessions with the intervention format in the first half, B in the second half. Between-group identity stages and self-regulated learning levels were compared using professional identity essays and the Motivated Strategies for Learning Questionnaire.” | “Two-level regression analyses showed no improvement in questionnaire categories but moderate improvement of professional identity stages over time (R2 = 0.069), regardless of timing of intervention.” | “Professional identity moderately forms during the pre-clinical year curriculum. However, neither identity nor self-regulated learning is raised significantly by limited intervention.” |
| McLachlan 2010 | **Measuring conscientiousness and professionalism in**  **undergraduate medical students** | Qualitative | NA | 37-38 | We are interested in hints from the literature that a significant part of professionalism is what might be thought of as diligence or conscientiousness.” | “We award students points on every occasion when they might be conscientious in performing simple tasks (such as attending compulsory sessions, providing essential documentation and participating in required administrative procedures). This is aggregated over the year to give a continuous, objective and multi-occasion score that is inexpensive to construct. We then determine the relationship of this score with independent staff and student estimates of professionalism.” | “We observe a positive correlation between conscientiousness and professionalism at both high and low ends of the spectrum.” | “This correlation raises a number of further questions. What is the sensitivity and specificity of this measure? How might it best be used with students: as a formative tool to change behaviours or as a summative tool to affect progression? And how will students react to its use? Finally, can it be extended to spheres other than undergraduate education, for instance with postgraduate trainees?” |
| McLachlan et al. 2009 | **The Conscientiousness Index: A Novel Tool to Explore Students’ Professionalism** | Qualitative | NA | 559-564 | “Measuring professional behavior is problematic not least because the concept of professionalism is difficult to define. The authors describe a measurement tool that does not rely on qualitative judgments from respondents but, nonetheless, clearly correlates with individuals’ subjective views about what constitutes professional behavior.” | “The authors devised the Conscientiousness Index (CI) of medical students’ performance in years 1 (n 116) and 2 (n 108) in 2006 –2007. The CI scores were based on a range of objective measures of conscientiousness, including attendance and submission of required information (such as immunization status or summative assessments) by a deadline. The validity of this instrument was tested against (1) staff views of the professional behavior of individual students and (2) critical incident reports.” | “The trait of conscientiousness, as measured by the CI, showed good correlation with the construct of professionalism as perceived by staff views of individual students’ professional behavior. There was also a relationship with the frequency of critical incident reporting. Together, these observations support the validity of the approach. Reliability and practicality were also acceptable.” | “The results suggest that the CI measures a scalar objective trait that corresponds well with professional behavior as perceived by staff members in an undergraduate medical school. The individual decisions making up the CI are objective and easy to collect, making it a relatively simple and uncontroversial method for exploring students’ professionalism.” |
| Mueller 2008 | **Incorporating Professionalism into Medical Education:**  **The Mayo Clinic Experience** | Descriptive |  |  | “In this article, the statements of profes- sional societies (e.g., the Charter on Medical Professionalism), the expectations of patients and society regarding professionalism, and a framework for defining medical professionalism are described” |  |  | "Indeed, the Mayo Clinic experience validates professionalism  as a core physician competency.” |
| Norcini & Shea 2016 | **Assessment of professionalism and progress in the development**  **of a professional identity** | Perspective | NA | NA | “This chapter will address the assessment of professionalism by (1) outlining the challenges, (2) citing reasons for assessing it, (3) using Miller’s pyr- amid as a framework for describing some of the methods of assessing professionalism and the research that supports them, and (4) suggesting some principles for developing an assessment system for professionalism. We conclude with brief consid- eration of lessons learned and future directions.” | NA | NA | “The assessment of professionalism offers the pos- sibility to make a positive difference in the training of physicians, the accountability of the profession, and the quality of patient care. For doctors, it offers a route to the development of professional identity and the identification of strengths and weaknesses. For the profession, it can fulfill an obligation to society. For patients, it provides a safeguard that their doctors will treat them in the way that they deserve. But as Colton warns us, it is important that we use them in a wise way.” |
| O’Tuathaigh et al. 2019 | **Medical students’ empathy and attitudes towards professionalism: Relationship with personality, specialty preference and medical programme** | Quantitative | 13.5 | NA | “This study was designed to explore the putative association between personality, psychometrically measured empathy, and attitudes towards medical professionalism in medical students as measured by a validated tool which focuses on four aspects of medical professionalism (personal characteristics; interaction with patients; social responsibility; interactions with the health). We also sought to investigate further the association between empathy and personality (as measured in the NEO-FFI) in an Irish medical school sample. Lastly, we sought to determine and compare empathy scores according to gender, year of study, mode of entry (direct-entry, graduate-entry), and career specialty preferences.” | “A cross-sectional questionnaire-based study was conducted in first and final year medical students at an Irish medical school. The following instruments were administered: (a) Jeffer- son Scale of Empathy; (b) NEO Five-Factor Inventory (NEO-FFI-3); (c) Attitudes towards Professionalism Scale. Demographic and educational variables were also measured. Descriptive and correlational analysis was conducted to examine the association between empathy, personality, professionalism-related attitudes and additional measures. Regres- sion analysis was used to examine determinants of attitudes towards professional behaviour.” | “Both selected NEO-FFI personality traits and empathy were independently associated with distinct categories of professional behaviour. Specifically, Openness to Experience was associated with higher empathy scores, and higher ‘Social responsibility’. Extraversion was linked with higher scores on the “Personal characteristics” and “Interactions with team” categories, while Conscientiousness was also positively associated with “Personal characteristics”. In agreement with previous studies, the personality traits most associated empathy were Agreeableness and Openness to Experience. Empathy did not vary according to programme year or career specialty preference” | “This study is the first to show that empathy and personality factors may act as determinants of students’ attitudes towards medical professionalism in a manner which is dependent upon category of professional behaviour.” |
| Olive & Abercrombie 2017 | **Developing a Physician's Professional Identity Through Medical Education** | Perspective | NA | NA | “Teaching and assessing the knowledge of professionalism's nature, history and purpose remains necessary, but additional attention should be given to the process of PIF.” | NA | NA | “To ensure professional behavior and adequate PIF in future physicians, appropriate measures should be taken to intentionally place opportunities for reflection and normative justification in the curriculum by focusing on sentinel events. Appropriate assessment tools are necessary to demonstrate that the learner has achieved competency, and the tool should be appropriate for the learner and the prescribed learning environment. Many unanswered questions remain surrounding remediation and publication of outcomes-based interventions is needed.” |
| Ong et al. 2015 | **The sociocultural and ethical issues behind the decision for artificial hydration in a young palliative patient with recurrent intestinal obstruction** | Perspective | NA | NA | “To highlight the complex interplay of ethical, clinical, practical, and psychosocial considerations behind such a decision, we discuss the decision-making process behind the determination to commence AH for a young 24-year-old Chinese woman with progressive metastatic ovarian adenocarcinoma who maintained a good functional status despite recurrent episodes of intestinal obstructions.” | NA | NA | “In conclusion, the approach to AH should be individualised to the patient’s needs. AH at home is feasible with good family support and a home hospice team. However, it should not be viewed as a means of prolonging survival, and both patient and family must understand the possible complications before the initiation of AH.” |
| Ottenberg et al. 2015 | **An analysis of reflective writing early in the medical curriculum: The relationship between reflective capacity and academic achievement** | Mixed methods | 12 | 724-728 | “To examine the relationship between reflection, gender, residency choice, word count, and academic achievement among medical students.” | “A modified version of the Reflection Evaluation for Learners’ Enhanced Competencies Tool (REFLECT) was developed and used for this study (Cronbach’s alpha of 0.86 with an intraclass correlation coefficient [ICC] of 0.68). This was applied to writing samples about professionalism in gross anatomy from first-year medical students between 2005 and 2011. Four analysts reviewed and scored written reflections independently. Composite reflection scores were compared with gender, residency choice, length of written reflection, NBME Gross Anatomy and Embryology Subject Examination scores, and final gross anatomy course.” | “Total of 319 written reflections were evaluated. Female students who pursued medicine specialties had the highest composite reflection scores (87 [27.2%]). Word count frequently correlated with reflection score (p50.0001). Students who performed well on the NBME Gross Anatomy and Embryology Subject Examination tended to achieve high anatomy course grades (p50.0001). There was no statistically significant relationship between composite reflection scores and NBME Gross Anatomy and Embryology Subject Examination scores (p 1⁄4 0.16) or anatomy course grades (p 1⁄4 0.90).” | “This study suggests that there are no preliminary correlations between reflective capacity and academic achievement; nevertheless, the value of reflection should not be discounted. Reflection is still important for building skills around critical thinking, self-evaluation, stress management, and meaning making. Knowing how to pair evidence with story is what will enable our future doctors to provide quality care to patients and to develop creative solutions for the future. Moving forward, more research should be completed around how to evaluate reflection and how best to respond to students with low reflective capacity in an effort to encourage professional success. In addition, discussion should continue around what skills are most valued among medical students and whether nontechnical skills should be more rewarded.” |
| Pangaro 2006 | **A Shared Professional Framework for Anatomy and Clinical Clerkships** | Viewpoint | NA | NA | “This essay discusses alternative educational frameworks in which professionalism can be located. As the traditional analytic framework (knowledge, skills, and attitudes) and developmental frameworks are more familiar, emphasis will be placed on a ‘‘synthetic’’ framework that expresses a student’s progress as ‘‘reporter,’’ ‘‘interpreter,’’ and ‘‘manager/educator.’’” | NA | NA | “This ‘‘RIME’’ framework attempts to capture the classic rhythm of observation–reflection–action that is familiar to all scientists and clinicians, and attempts to express in less generic, more behavioral terms how skills, knowledge, and attitudes must all be brought to bear at the same time by a successful student. It is argued that the complexity of professional development can be embraced with simplicity, without being simplistic.” |
| Parker et al. 2008 | **The “Pyramid of Professionalism”: Seven Years of Experience With an Integrated Program of Teaching, Developing, and Assessing Professionalism Among Medical Students** | Descriptive | 9 | NA | “The authors report on an integrated program of teaching, developing, and assessing professionalism as well as managing unprofessional behavior referrals and supporting students through the Personal and Professional Development Committee (PPDC) in the four-year, graduate-entry medical program at the School of Medicine, University of Queensland, Australia.” | “Two thousand six hundred thirty medical students have participated in the ethics and professional practice teaching program from 2000 to 2006. They were assessed through formal examination; students who did not satisfy requirements completed supplementary examinations.” | “One student failed a year on the basis of formal examination. Instructors referred 507 students (19% of all enrolled) during the seven-year period to the PPDC, which interviewed 142 (25%; 5% of all enrolled) at least once; 25 of these more than once. In all, 711 reports were submitted to the PPDC, 420 (55%) for unsatisfactory attendance only and 291 (45%) for other concerns. Most of these (51%) related to “responsibility/reliability” and “participation” combined; 12% related to “honesty,” “discrimination,” and “doctor–patient relationship.” The PPDC referred four students to the board of examiners, and two students failed a year for persistent unprofessional behavior.” | “The authors established a Pyramid of Professionalism whose foundation is a formal curriculum of medical ethics, law, and professionalism. At higher levels, the pyramid mirrors Australia’s medical regulatory processes, combining nonpunitive support with the possibility of sanctions, by mediating and sometimes remediating a range of notified concerns. Students who persist in behaving unprofessionally or in seriously unacceptable ways have failed academically on professionalism grounds.” |
| Pratt et al. 2020 | **Constructing Professional Identity: The Role of Work and Identity Learning Cycles in the Customization of Identity among Medical Residents** | Qualitative | NA | 239-262 | “The purpose of this article is to build and enrich theory around how professionals construct their own professional identities. We examined this pro cess in the context of medicine, as it is thought to be a prototypical profession. Specifically, we examined physicians during residency training, which is thought to "contribute to the development of commitment to the occupation as a life career and to a shared identity, a feeling of community or solidarity among all those who have passed through it". Thus, un like sociological treatments exploring how professions as an occupational group establish legitimacy, status, and boundaries, the present treatment focused on individual actors.” | “Through a six-year qualitative study of medical residents, we build theory about professional identity construction.” | “We found that identity construction was triggered by work-identity integrity violations: an experienced mismatch between what physicians did and who they were. These violations were resolved through identity customization processes (enriching, patching, or splinting), which were part of interrelated identity and work learning cycles. Implications of our findings (e.g., for member identification) for both theory and practice are discussed.” | “First, our research shows how each of several existing bodies of knowledge (including socialization, career/role transitions, and identity work) illuminates separate aspects of identity construction. Additionally, in our findings and in our construction of a theoretical model, we make several advances in understanding professional identity construction.” |
| Roberts & Stark 2008 | **Readiness for self-directed change in professional behaviours: factorial validation of the Self-reflection and Insight Scale** | Quantitative | 9.5 | NA | “The aim of this study in exploring the utility of the SRIS for measuring students’ readiness for making purposeful self-regulated changes in professional behaviour was threefold.” | “We used structural equation modelling to undertake a confirmatory factor analysis of the SRIS. We re-specified our model to analyse all of the data to explain relationships between the SRIS, medical student characteristics, and responses to issues of teaching and learning in professionalism.” | “The factorial validity of a modified SRIS showed all items loading significantly on their expected factors, with a good fit to the data. Each subscale had good internal reliability (> 0.8). There was a strong relationship between the need for reflection and engagement in reflection (r = 0.77). Insight was related to need for reflection (0.22) and age (0.21), but not to the process of engaging in reflection (0.06).” | “Validation of the SRIS provides researchers with a new instrument with which to measure and investigate the processes of self-reflection and insight in the context of students’ self-regulation of their professionalism. Insight is related to the motive or need for reflection, but the process of reflection does not lead to insight. Attending to feelings is an important and integral aspect of self-reflection and insight. Effective strategies are needed to develop students’ insight as they reflect on their professionalism.” |
| Roberts et al. 2017 | **Peer assessment of professional behaviours in problem-based learning groups** | Quantitative | 10 | NA | “We investigated whether a peer assessment of learning behaviours in PBL is sufficiently valid to support decision making about student professional behaviours.” | “Data were available for two cohorts of students, in which each student was rated by all of their PBL group peers using a modified version of a previously validated scale. Following the provision of feedback to the students, their behaviours were again peer-assessed. A generalisability study was undertaken to calculate the students’ professional behaviour scores, sources of error that impacted the reliability of the assessment, changes in student rating behaviour, and changes in mean scores after the delivery of feedback.” | “Peer assessment of professional learning behaviour was highly reliable for within-group comparisons (G = 0.81–0.87), but poor for across-group comparisons(G = 0.47–0.53). Feedback increased the range of ratings given by assessors and brought their mean ratings into closer alignment. More of the increased variance was attributable to assessee performance than to assessor stringency and hence there was a slight improvement in reliability, especially for comparisons across groups. Mean professional behaviour scores were unchanged.” | “Peer assessment of professional learning behaviours may be unreliable for decision making outside a PBL group. Faculty members should not draw conclusions from peer assessment about a student’s behaviour compared with that of their peers in the cohort, and such a tool may not be appropriate for summative assessment. Health professional educators interested in assessing student professional behaviours in PBL groups might focus on opportunities for the provision of formative peer feedback and its impact on learning.” |
| Sattar et al. 2016 | **Your professionalism is not my professionalism: congruence and variance in the views of medical students and faculty about professionalism** | Quantitative | 8.5 | NA | “Medical professionalism is an essential aspect of medical education and practice worldwide and it must be adopted according to different social and cultural contexts. We examined the current congruence and variance in the perception of professionalism in undergraduate medical students and faculty members in one medical school in Saudi Arabia.” | “The target population was first year to final year medical students of College of Medicine, King Saud University. Out of a total of 1431 students at College of Medicine 750 students (52 %) participated in the study. Fifty faculty members from clinical and non-clinical departments of the College of Medicine were randomly selected for this study and all participated in the study. The respondents recorded their responses through the Bristol online survey system, using a bilingual (English and Arabic) version of the Dundee Polyprofessionalism Inventory I: Academic integrity, which has 34 items.” | “There are 17 lapses (50 % of the total) in professional behaviour where none of the faculty recommend the ignore sanction while students recommended a variable ignore sanction in a range of 6–29 % for different behaviours. Students and faculty recommended similar sanctions for 5 lapses (14.7 % of the total) in professional behaviours. Furthermore, there is statistically significant two level difference between the sanctions approved by faculty and students in the recommended sanctions for 12 lapses (35 % of the total (p < 0.050).” | “These results raised concerns in relation to the students’ understanding of professionalism. It is therefore, important to enhance their learning around the attributes of medical professionalism.” |
| Schei et al. 2019 | **Reflection in medical education: intellectual humility, discovery, and know-how** | Perspective | NA | NA | “We argue that knowledge of mechanisms underlying human thought may be useful in designing educational programs to foster desired attributes such as curiosity, critical self-awareness and intuitive acumen in medical professionals. The juxtaposition of neuroscientific insights with ideas from Kant on reflective judgement, van Manen on tact, and Aristotle on phronésis, supports a concept of reflection that manifests as wise practice.” | NA | NA | “We suggest that reflection in medical education should be (a) an imperative for educators seeking to guide learners to man- age the complexity and “messiness” of medical practice, and (b) a role-modelling mode of medical practice characterized by self-correcting behaviors that culminate in good and right professional actions. An example illustrates reflective practice in the teaching and learning of physicianship.” |
| Steinert et al. 2007 | **Faculty Development as an Instrument of Change: A Case Study on Teaching Professionalism** | Descriptive | NA | 1057-1063 | “The authors describe that program to illustrate how faculty development can serve as a useful instrument in the process of change.” | NA | NA | “Faculty development has clearly been a powerful instrument of change in our setting, and we hope that other programs can benefit from some of our lessons learned.” |
| Tagawa 2019 | **Development of a scale to evaluate medical professional identity formation** | Mixed methods | 10.5 | 2-9 | “Medical educators now focus on professional identity formation (PIF), which is a process of psychological development and socialization in the community of practice. This study aimed to develop an instrument to evaluate PIF that can be applied to a large group of medical trainees.” | “A self-administered questionnaire was created with items on priorities, behavior standards, attitudes, and emotional control of well-developed physicians, in addition to items on their background and experience in playing the role of a physician. The participants were divided into four respondent groups: 4th- and 6th-year medical students and 2nd-year residents at Kagoshima University, and experienced medical doctors (instructors).” | “Using factor analysis of data from 318 respondents and respondent group comparison, a developing scale (DS) with 15 items was created. The DS has a five-factor structure and evaluates self-control as a professional (factor 1), awareness of being a medical doctor (factor 2), reflection as a medical doctor (factor 3), execution of social responsibility (factor 4), and external and internal self-harmonization (factor 5). The mean DS score of the instructors was significantly higher than that of the residents (p < 0.01), the mean score of residents and instructors was significantly higher than that of students (p < 0.01), and the mean score of instructors was significantly higher than that of all other respondents (p < 0.01). Respondent group, but not gender, was a significant variable of the DS. The DS and scores of factors 2 and 4 correlated with 6th-year medical students’ experience in playing the role of a physician during clinical training, and scores of factors 3 and 4 correlated with 2nd-year residents’ experience in playing the role of a physician. There was no significant difference between the mean DS score of 4th- and 6th-year medical students, which might due to less clinical experience among 6th-year medical students or a limitation of the scale to evaluate pre-clinical medical students.” | “The DS could be a useful indicator of medical trainees’ personal and professional development and socialization. Experience in playing the role of a physician might facilitate medical trainees’ PIF.” |
| Tagawa 2020 | **Scales to evaluate developmental stage**  **and professional identity formation in medical students, residents, and**  **experienced doctors** | Mixed methods | 10.5 | 2-9 | “I proposed a development scale (DS) to quantitatively evaluate the degree of maturation and socialization as a physician; however, one scale is not enough to illustrate the helical and complex process of development.” | “Using Kegan’s model as the conceptual framework, scales that evaluate stage 2, 3, and 4, and higher stage-specific attributes were developed using data collected in a self-administered questionnaire (322 respondents), reliability analysis, group comparison, and analysis of individual DS scores. The respondents were 4thand 6th-year medical students and 2nd-year residents at Kagoshima University, and experienced medical doctors (instructors).” | “In addition to the DS, one self-administered questionnaire consisting of 27 items for stage 2, 3, 4, and higher stage-specific attribute scales was created. Students had the highest mean score in stage 2, and instructors had the highest mean score in stage 4 and higher stage scales. Individual analysis indicated that there were respondents with varied attributes in each group, that the average medical student might have inclusion preference typically seen at stage 3, and that the average instructor might have independent preference typically seen at stage 4 more than inclusion preference.” | “Combining multiple stage attribute-specific scales and DS scores could quantify the complexity and divergent processes of PIF. These scales could provide meaningful information about individuals, groups, and education in terms of professional development that is different from assessment data of medical knowledge or professional skills.” |
| Tay et al. 2020 | **Assessing Professionalism in Medicine – A Scoping Review of Assessment Tools from 1990 to 2018** | Systematic review | NA | NA | “The absence of such a context-specific, culturally appropriate and linguistically sensitive assessment tool within the Singapore setting inspired this scoping review. It is hoped that the insights proffered will guide design of a tool for the local context.” | “Arksey and O’Malley’s (2005) approach to scoping reviews was used to identify appropriate publications featured in four data- bases published between 1 January 1990 and 31 December 2018. Seven members of the research team employed thematic analysis to evaluate the selected articles.” | “3799 abstracts were identified, 138 full-text articles reviewed and 74 studies included. The two themes identified were the con- text-specific nature of assessments and competency-based stages in medical professionalism.” | “Prevailing assessments of professionalism in medicine must contend with differences in setting, context and levels of pro- fessional development as these explicate variances found in existing assessment criteria and approaches. However, acknowledging the significance of context-specific competency-based stages in medical professionalism will allow the forwarding of guiding principles to aid the design of a culturally-sensitive and practical approach to assessing professionalism.” |
| Van mook et al. 2010 | **Combined formative and summative professional behaviour assessment approach in the bachelor phase of medical school: A Dutch perspective** | Descriptive | NA | NA | “Although, many examples of frameworks of professionalism and PB can be found in the literature, most originate from North America, and only few are designed in other continents. This article presents the framework for PB that is used at Maastricht medical school, the Netherlands.” | NA | “The approach to PB used in the Dutch medical schools is described with special attention to 4 years (2005–2009) of experience with PB education in the first 3 years of the 6-year undergraduate curriculum of Maastricht medical school. Future challenges are identified.” | “The adages ‘Assessment drives learning’ and ‘They do not respect what you do not inspect’. Professionalism in medical education, an American perspective: From evidence to accountability. suggest that formative and summative aspects of PB assessment can be combined within an assessment framework. Formative and summative assessments do not represent contrasting but rather complementary approaches. The Maastricht medical school framework combines the two approaches, as two sides of the same coin.” |
| Van Mook et al. 2012 | **Web-assisted assessment of professional behaviour in problem-based learning: more feedback, yet no qualitative improvement?** | Mixed methods | 10 | 83-86 | “This study has two research goals: it focuses on the quantity and quality of comments provided by students and their peers (two researchers independently scoring comments as correct and incorrect in relation to five commonly used feedback rules (and resulting in an aggregated score of the five scores) on the one, and on the feasibility, acceptability and perceived usefulness of the two approaches on the other hand (using a survey).” | “The study involved all medical students enrolled in the second, ten-week course in year 2 at the Faculty of Health, Medicine and Life Sciences, Maastricht University, the Netherlands. During the bachelor programme of the six-year problem-based medical curriculum, professional behaviour is assessed on various occasions in tutorial groups during all regular courses. Each tutorial group consists of ten students on average and a tutor/facilitator, and each meeting lasts 2 h. For the purpose of this study, the students were divided into two groups: those in tutorial groups with even numbers and those in groups with odd numbers. The first group used a web-based instrument to assess professional behaviour and the other group used the usual method with a paper assessment form.” | “The amount of feedback was significantly higher in the web-based group than in the paper based group for all three categories (dealing with work, others and oneself). Regarding the quality of feedback, the aggregated score for each of the three categories was not significantly different between the two groups, neither for the interim, nor for the final assessment. Some, not statistically significant, but nevertheless noteworthy trends were nevertheless noted. Feedback in the web-based group was more often unrelated to observed behaviour for several categories for both the interim and final assessment. Furthermore, most comments relating to the category ‘Dealing with oneself’ consisted of descriptions of a student’s attendance, thereby neglecting other aspects of personal functioning.” | “The survey identified significant differences between the groups for all questionnaire items regarding feasibility, acceptability and perceived usefulness in favour of the paper-based form. The use of a web-based instrument for professional behaviour assessment yielded a significantly higher number of comments compared to the traditional paper-based assessment. Unfortunately, the quality of the feedback obtained by the web-based instrument as measured by several generally accepted feedback criteria did not parallel this increase” |
| Wald 2015 | **Refining a definition of reflection for the being as well as doing the work of a physician** | Perspective | NA | NA | “Medical educators thus grapple with operationalizing and effectively integrating reflection as a foundational construct within health care professions education and practice.” | “Core elements of reflection including role of emotions and awareness of self, other and situation, do not appear within various working definitions of reflection.” | “This observation as well as noted recent shift in medical education toward emphasis on the ‘‘being’’ as well as ‘‘doing the work’’ of a physician led to the author’s proposed refining of Sandars’ reflection definition and expansion of Nguyen et al.’s reflection model.” | “A refined reflection definition is offered for a more inclusionary approach. A caveat regarding potential for expected reflective learning outcomes (given reflection as a process) is provided and the integral role of mentor-enhanced reflection is discussed. Reflection as a continuum is highlighted and exemplified within Wald et al.’s REFLECT rubric and Nguyen et al.’s reflection model.” |
| Wald 2015 | **Professional Identity (Trans)Formation in Medical Education: Reflection, Relationship, Resilience** | Commentary | NA | NA | “The author of this Commentary provides an overview of foundational principles and key drivers of PIF supporting the being, relating, and doing the work of a compassionate and competent physician.” | NA | NA | “Outcomes assessment can be challenging. Descriptive, formative assessment using mixed methods to provide feedback, evaluate curricular programs, and guide theoretical development is recommended by the TIME task force.” |
| Wald et al. 2009 | **Reflecting on Reflections: Enhancement of Medical Education Curriculum With Structured Field Notes and Guided Feedback** | Qualitative | NA | 830-832 | “The development and implementation of this innovation are presented, as is an analysis of the written evaluative comments of students taking the Doctoring course. Theoretical and clinical rationales for features of the innovation and supporting evidence of their effectiveness are presented.” | “We conducted qualitative content analyses of the students’ responses for theme extraction. Successive rounds of individual and group analyses used “immersion-crystallization,” a qualitative analytic style involving cycles of concentrated textual review of data, combined with reflection and intuitive insights, until reportable interpretation becomes apparent.” | “Qualitative analyses of students’ evaluations yielded four themes of beneficial contributions to their learning experience: promoting deeper and more purposeful reflection, the value of (interdisciplinary) feedback, the enhancement of group process, and personal and professional development. Evaluation of the innovation was the fifth theme; some limitations are described, and suggestions for improvement are provided. Issues of the quality of the educational paradigm, generalizability, and sustainability are addressed.” | “In general, the results of the pilot evaluation highlight successful implementation of this innovative method of structured field notes and guided feedback within medical education, which is consistent with the theoretical framework and stated goals of promoting reflection and fostering professionalism. We propose this curriculum innovation as an incremental step forward within medical education toward the realization of such goals.” |
| Wald et al. 2010 | **‘The Loss of My Elderly Patient:’ Interactive**  **reflective writing to support medical students’**  **rites of passage** | Descriptive | NA | E179-E182 | “At Warren Alpert Medical School of Brown University (Alpert Med), a narrative medicine curriculum innovation of students’ reflective writing (field notes) with individualized feedback from an interdisciplinary faculty team (in pre-clinical years) has been implemented in a Doctoring course to cultivate reflective capacity, empathy, and humanism. Interactive reflective writing (student writer/faculty feedback provider dyad), we propose, can additionally support students with rites of passage at critical educational junctures." | “At Alpert Med, we have devised a tool to guide faculty in crafting quality feedback, i.e. the Brown Educational Guide to Analysis of Narrative (BEGAN) which includes identifying students’ salient quotes, utilizing reflection-inviting questions and close reading, highlighting derived lessons/key concepts, extracting clinical patterns, and providing concrete recommendations as relevant.” | “We provide an example of a student’s narrative describing an emotionally powerful and meaningful event – the loss of his first patient – and faculty responses using BEGAN.” | “The provision of quality feedback to students’ reflective writing – supported by BEGAN – can facilitate the transformation of student to professional through reflection within medical education.” |
| Warm et al. 2017 | **Improving Learner Handovers in Medical Education** | Perspective | NA | NA | “To achieve these goals, the authors propose using a learner handover process modeled after a patient handover process.” | NA | NA | “Just as studies have evaluated patient handover models using metrics that matter most to patients, studies must evaluate this learner handover model using metrics that matter most to providers, patients, and learners.” |
| Weissbecker et al. 2016 | **Tracking Medical Professionalism Across the Educational Continuum** | Perspective | NA | NA | “Professionalism is a competency that can be shaped and developed over time; therefore, assessing behavior and growth across the educational continuum is essential. At Tulane, we are piloting a system that tracks patterns of behavior across all courses and years.” | NA | NA | “This approach allows for longitudinal assessment of student performance and helps identify those who need additional support to meet expected competencies. Moreover, maintaining a professionalism theme throughout the curriculum raises awareness of our commitment to a culture of professionalism and assures that our graduating students embody the standards and ethics essential to the practice of medicine.” |
| Wilkinson et al. 2019 | **A Blueprint to Assess Professionalism: Results**  **of a Systematic Review** | Systematic review | NA | NA | “In this study, conducted during 2007–2008, the authors aimed to match assessment tools to definable elements of professionalism and to identify gaps where professionalism elements are not well addressed by existing assessment tools.” | “The authors conducted literature reviews of definitions of professionalism and of relevant assessment tools, clustered the definitions of professionalism into assessable components, and clustered assessment tools of a similar nature. They then created a “blueprint” whereby the elements of professionalism are matched to relevant assessment tools.” | “Five clusters of professionalism were formed: adherence to ethical practice principles, effective interactions with patients and with people who are important to those patients, effective interactions with people working within the health system, reliability, and commitment to autonomous maintenance / improvement of competence in oneself, others, and systems. Nine clusters of assessment tools were identified: observed clinical encounters, collated views of coworkers, records of incidents of unprofessionalism, critical incident reports, simulations, paper-based tests, patients’ opinions, global views of supervisor, and self-administered rating scales.” | “Professionalism can be assessed using a combination of observed clinical encounters, multisource feedback, patients’ opinions, paper-based tests or simulations, measures of research and/or teaching activities, and scrutiny of self- assessments compared with assessments by others. Attributes that require more development in their measurement are reflectiveness, advocacy, lifelong learning, dealing with uncertainty, balancing availability to others with care for oneself, and seeking and responding to results of an audit.” |
| Wong & Trollope-Kumar 2014 | **Reflections: an inquiry into medical students’ professional identity formation** | Qualitative | NA | 3-4 | “The purpose of this study was to understand the major influences on medical students’ professional identity formation.” | “Sixty-five medical students (46 women; 19 men) from a class of 194 consented to the study of their portfolios. In total, 604 reflections were analysed and coded using thematic narrative analysis. The codes were merged under subthemes and themes. Common or recurrent themes were identified in order to develop a descriptive framework of professional identity formation. Reflections were then analysed longitudinally within and across individual portfolios to examine the professional identity formation over time with respect to these themes.” | “Five major themes were associated with professional identity formation in medical students: prior experiences, role models, patient encounters, curriculum (formal and hidden) and societal expectations. Our longitudinal analysis shows how these themes interact and shape pivotal moments, as well as the iterative nature of professional identity from the multiple ways in which individuals construct meaning from interactions with their environments.” | “Our study provides a window on the dynamic, discursive and constructed nature of professional identity formation. The five key themes associated with professional identity formation provide strategic opportunities to enable positive development. This study also illustrates the power of reflective writing for students and tutors in the professional identity formation process.” |
| Yakov et al. 2020 | **Mechanisms involved in the formation of professional identity by medical students** | Qualitative | NA | 1-5 | “To outline some major mechanisms involved in formation of medical students’ professional identity.” | “A qualitative study based on thematic analysis. 296 diary entries, written by eight medical students as part of the three-year course ’Becoming a Physician,’ were reviewed. The course, conducted in small groups, gradually exposes students to the clinical field, and emphasizes awareness to marginalized populations, and interpersonal communication skills.” | “Following the social constructivist learning theory, where learners individually and socially construct meaning attributed to their experiences, we identified three major mechanisms based on students’ written reflections. These include linking current experiences to past; comparing different types or aspects of knowledge, doctors or medicine; and future perspective taking.” | “This study sheds light on the possible mechanisms used by medical students in forming their professional identity prior to their clinical training. Knowing these mechanisms can help medical educators better understand and support their students in formation of their professional identities. Conducting reflective writing could serve as an effective tool for learners to comprehend the meanings of their experiences.” |
| Zhou et al. 2021 | **A systematic scoping review of approaches to teaching and assessing empathy in medicine** | Systematic review | NA | NA | “Empathy is pivotal to effective clinical care. Yet, the art of nurturing and assessing empathy in medical schools is rarely consistent and poorly studied. To inform future design of programs aimed at nurturing empathy in medical students and doctors, a review is proposed.” | “This systematic scoping review (SSR) employs a novel approach called the Systematic Evidence Based Approach (SEBA) to enhance the reproducibility and transparency of the process. This 6-stage SSR in SEBA involved three teams of independent researchers who reviewed eight bibliographic and grey literature databases and performed concurrent thematic and content analysis to evaluate the data.” | “In total, 24429 abstracts were identified, 1188 reviewed, and 136 included for analysis. Thematic and content analysis revealed five similar themes/categories. These comprised the 1) definition of empathy, 2) approaches to nurturing empathy, 3) methods to assessing empathy, 4) outcome measures, and 5) enablers/barriers to a successful curriculum.” | “Nurturing empathy in medicine occurs in stages, thus underlining the need for it to be integrated into a formal program built around a spiralled curriculum. We forward a framework built upon these stages and focus attention on effective assessments at each stage of the program. Tellingly, there is also a clear need to consider the link between nurturing empathy and one’s professional identity formation. This foregrounds the need for more effective tools to assess empathy and to better understand their role in longitudinal and portfolio based learning programs.” |
