# Supplementary material for: Assessing professional identity formation (PIF) amongst medical students in Oncology and Palliative Medicine postings: a SEBA guided scoping review
Source: BMC Palliat Care. 2022 Nov 18;21:200. doi: 10.1186/s12904-022-01090-4 (PMC9673314; doi:10.1186/s12904-022-01090-4)
Supplement: Supplementary file 3 — Additional file 3: Appendix C. Stage-based nature of PIF. [file 12904_2022_1090_MOESM3_ESM.docx]

Appendix C. Stage-based nature of PIF

##### Knows and knows how

| Levels of amended Miller’s pyramid | Knowledge assessed | Forms/ tools of assessment |
| --- | --- | --- |
| ‘Know’ and ‘knows how’ | - Legal knowledge (113, 127) - Professionalism-related knowledge (113, 127) - Ethical Knowledge (68, 113, 114) - Narrative competence (68) - Moral reasoning (114) | Traditional assessment methods (95, 113, 114)   - MCQ (95, 104, 114, 121) - SAQ (121) - Objective structured video examinations (104) - OSCEs (95, 113). |
| ‘Know’ | Professionalism-related knowledge (113, 127) | Barry Challenges the Professionalism Questionnaire (114)   - Patient-based items: Conflicts of interest, Confidentiality, Physician Impairment, Honesty, Sexual harrassment, Acceptance of Gifts   Critical Incident reflection (104, 114)  Tenets (values) of professionalism involved in critical incidents (114) |
|  | Ethical Knowledge (68, 113, 114) | Test of Residents’ Ethics Knowledge for Pediatrics (TREK-P) (114) |
| ‘Knows how’ | Moral reasoning (114) | Moral judgement interview (114)  Defining Issues Test (104, 114, 120, 127) |
|  | Professionalism-related knowledge (113, 127) | Situational Judgement Test (114)  Critical incidents (104, 114) |

##### Shows how/ does

| Types of behaviour and its intentions | | | | Tools of assessment |
| --- | --- | --- | --- | --- |
| Types of behaviour | | | |  |
| Interpersonal | Honesty and integrity | General | Communicating honestly and empathetically (29) | - Written exams (73) - Professional behaviour assessment form (103) - Completion of tasks/ attendance at activities (68, 92, 100, 102, 103) - Directly observed behaviour   - Mini-CEX (109)   - In-training evaluation reports (136)   - Ophthalmic clinical evaluation exercise (104)   - Standardised direct observation tool (104) - Behaviour observed in OSCE (68, 107, 114, 121) - Revised global assessment form (99) - Ethical dilemmas in high-fidelity patient simulations(104) - Online behaviour (92) - Paper-based   - Critical incident reports (61, 73, 103, 104, 107)   - Incident report form (104)   - Defining issues test (104)   - Objective structured video examination (104)   - Multiple-choice test (104) - Survey (68, 128) - Web-based assessment (132) - Physicianship evaluation form (109) - Open-ended feedback (111) - Professionalism mini-evaluation exercise (62, 99, 106, 109, 114, 133) - Conscientiousness index (92, 108, 114, 135) - Peer assessment instrument by Papinczak (126) - Professionalism checklist (109) - Multisource feedback (104) - Professionalism rubric (74) - Rating scales (61, 73) - Maastricht Peer Activity Rating Scale (126) - Structured assessment scale (111) - Patient opinion   - Feedback and Care Evaluation (FACE) cards (104, 128)   - Royal College of Physicians Patient Questionnaire (104)   - Wake Forest Physician Trust Scheme (104)   - Patient assessment questionnaire (PAQ) (104)   - Simulated patient rating scales (104)   - Humanism scale (104) - Global view of supervisor   - Global rating form (104)   - University of Michigan Department of Surgery (104)   - Professionalism Assessment Instrument (104)   - Evaluation of professional behaviour in general practice(EPRO- GP) (104)   - Amsterdam attitudes and communication scale (104) |
|  |  | Involving healthcare team | Report dishonesty amongst team (94) |  |
|  | Responsibility and participation | Involving peers | - Contributes willingly in team-based and community activities (29, 74) - Shares learning opportunity with peers (74) - Offers assistance to peers being prompted (74) |  |
|  |  | Involving healthcare team | - Offers assistance to team members without being prompted (74) - Takes initiative to alert team (74) - Supportive of health care team and contributes to a supportive environment in all team-based activities (74) |  |
|  |  | Involving patients and their families | - Prepares well before seeing each patient (74) - Meticulous/ Attention to detail (74) - Genuine interest in patient (62, 74) - Follow up to ensure proper care (74) - Ensured continuity of patient care(62) - Priorities the patients’ and families’ needs over self-interest or personal advantage (74) |  |
|  |  | Involving society (social responsibility) | - Committed to medical professionalism as a socially responsible force for the good (74) - Committed to serve others in need as a socially responsible professional (74) - Treating the underprivileged first (94) - Improve access to care (94) - Just distribution of resources (62, 94) - Teach and disseminate knowledge (94) - Advocate for patients (62, 94) - Manage conflicts of interest (94) |  |
|  | Respect and Sensitivity | General | - Avoid derogatory language (62) |  |
|  |  | Involving peers | - Pays attention, contributes appropriately, and does not distract others or engage them in conversation (74) |  |
|  |  | Involving patients and their families    Secondary: (Tay et al. 2020) | - Attentive, appropriately responsive and polite in all interactions (74) - Adequate nonverbal behaviour (103) - Patients concerns first (94) - Handling conflict (103) - Involve patients in decision making (29, 94, 104) - Ensures patient’s Confidentiality, privacy maintained; modesty protected (62, 68, 74, 94, 100, 103) - Good rapport before requesting information of private or personal nature (74) - Responsive to the needs of those accompanying patients (eg: friends/ relatives) (74) - Respects patients need (62, 74) - Accepts limits to student examination in interest of patient welfare (74) - Maintaining Professional Boundaries with patients (62, 74, 104) - Behave equitably towards all, irrespective of gender, age, culture, social and economic status, sexual identity (74) - Displays an awareness of the impact of financial issues for patients in clinical care (74) |  |
|  |  | Involving healthcare professionals | - Behaves within appropriate and safe limits in clinical settings (attire, etc) (62, 74, 94) - Actively seeks and values inputs from all Health care professionals in the team (74) - Maintaining professional boundaries between healthcare professionals (104) - Being supportive to team members (100, 111) |  |
|  | Compassion and Empathy | Involving peers | - Strives to understand fellow student’s needs and respond appropriately (74) |  |
|  |  | Involving patients and their families | - Strives to meet patient’s and families; physical and emotional needs (when appropriate) (74) - Listen Actively to patient (62) - Reflects on observed caring attitudes toward patients (74) - Demonstrated a caring attitude towards patients (feedback from peers and staff) (74) - Providing holistic care and showing interest in patient as a person (29) |  |
|  | Others | Involving healthcare professionals | - Bringing positive influence to the work environment (29) - Available to colleagues (62) |  |
|  |  | - Managing conflicts of interest (29) - Managing challenging situations/interactions (127) - Avoiding abuse of power (94) | |  |
| **Individual** | Honesty and integrity | - Admit errors and omissions (62) - Reliable and accurate histories and physical findings (74) - Does the correct thing (aware of being observed or not) (74) - Prioritises truth above self-interest (admits when action not taken) (74) - Accepts personal mistakes and honestly acknowledges them (74) | |  |
|  | Humility | - Seek and respond to feedback; Respond to error; Recognize limits (29, 62, 103, 104, 111) - Lifelong learning (29, 104) - Response to assessment (94) - Address own gaps in knowledge and skills (62) | |  |
|  | Responsibility and participation | - Fulfil commitment (74) - Punctuality (29, 62, 68, 74, 94, 100, 102, 103, 111) - Attendance (94) - Adequately prepared for agreed tasks (74) - Completing assigned tasks (62, 100) - Taking responsibilities (104) - Time management, organization (29, 104) - Clinical setting   - Follow formal policies (74)   - Practice within own limit (74) - Seeks help when needed (74) | |  |
|  | Striking balance | - Balance availability to others with care for oneself (104) - Establishing Personal-professional lifestyle balance, as well as time for scholarly pursuits (62) | |  |
|  | Others | - Dealing with uncertainty (104) - Asking Help when needed such as for mental and physical abuse (29) - Maintaining Composure in difficult situations (29, 62) - Adhering to and role modelling ethical conduct and professionalism (29, 94) | |  |
| Intentions behind behaviour | | | |  |
| Values being assessed from behaviours | Personal | - Excellence (93, 109) - Internal motivation (94) - Responsibility (128) - Being reliable (94) - Respect (111) - Self-improvement (94) - Honesty (74, 94) - Integrity (68, 74, 93, 100, 109) - Compassion (74, 100, 128) - Empathy (68, 74) - Conscientiousness (92, 94, 108, 109, 135) - Accountability (94, 109, 111) - Courtesy (100, 103) | |  |
|  | Interpersonal | Involving patients and their families | - Respect for patients (94, 103, 109, 128) - Respect for family (94) |  |
|  |  | Involving team (healthcare team / peers) | - Respect for others (62, 94, 100, 111) - Personal interactions (93) - Teamwork (109) |  |
|  | Others | - Personal relationships (109) | |  |
| Attitudes being assessed from behaviour | Personal | - pursuit of excellence/insight (93, 109) - Honouring commitments (103) - Commitment to learning/ participation (94, 111) - Stress tolerance (109) - Appropriate self-criticism (111) | |  |
| Knowledge being assessed from behaviour | Personal | - Knowledge of limits (94, 100) | |  |
|  | Society | - Social norms (109) - Context (109) - local cultures/ cultural differences(109) | |  |
| Skills being assessed from behaviour | Personal | - Self-awareness (100) - Self-care (100) - Self control (69) | |  |
|  | interpersonal | - Leadership (103) - Communication (62, 100, 103, 111, 128) | |  |
| Others being assessed from behaviour | Intentions | - Behavioural Intentions (109) | |  |

| Types of skills | | Assessment tools |
| --- | --- | --- |
| Social/ Interpersonal skills | Communication skills (29, 68, 95, 99, 114, 120, 127, 128, 130) | - Communications and Teamwork Skills (CATS) (68) - Simulated patients (104, 114) - Objective structured clinical examination (29, 68, 95, 99, 104, 127, 128, 130) - Introductory Clinical Experience (ICE) (120) - Communication skills checklist (120) - Multiple Mini Interview (120) - Simulated patients rating scale (104) – modification of Communications Skills Form developed at East Tennessee State University - Amsterdam attitudes and communication scales (104) - Reflective writing (29, 68, 95, 99, 127, 128, 130) - MCQs (29, 68, 95, 99, 127, 128, 130) - REFLECT rubric (29, 68, 95, 99, 127, 128, 130) - Professionalism workshops (128) - Dreyfus and Dreyfus Level of Master (29) - Millers performance level (29) - CLASS handover model (116) - Self-assessment (29) - Peer-assessment (29) - Multisource feedback (29) - Assessed by patients (29) |
|  | Teamwork skills (29, 68) | - Communications and Teamwork Skills (CATS) (68) - Peer evaluations (68) - Multiple Mini Interview(120) |
|  | Social awareness (128) |  |
|  | Leadership skills (29, 68, 104) | - Multisource feedback (104) - 360 degrees PPD judgement (104) |
|  | Interpersonal relationship skills (102, 104)   - Cooperative learning skills (102) | - Peer assessment (104) - Simulated patients rating scale (104) - Peer evaluation ratings (102) - Supervising physician assessment (102) - Dreyfus and Dreyfus Level of Master (29) - Millers performance level (29) |
|  | Doctor-patient relationship skills (29, 62, 104) | P-MEX (62, 104) |
|  | Interprofessional relationship skills (62, 104) | - Jefferson Scale MD-RN relationships (68) - P-MEX (62, 104) - Peer evaluations (68) - 360 evaluations (68) - Clerkship evaluation (68) - Simulated patients rating scale (104) |
| Personal | Reflection skills (29, 62, 68, 95, 104) | - Groningen reflection ability scales (104, 114) - P-MEX (62, 104) - Critical incident report (104) - REFLECT rubric (29, 68, 95, 99, 127, 128, 130) |
|  | Self-care (68) |  |
|  | Self-development (29) |  |
|  | Self-regulation (29, 99) |  |
|  | Self-awareness (29, 68, 128) | - Multiple Mini Interview (120) |
|  | Problem solving (29) | - Multiple Mini Interview (120) |
|  | Critical thinking (29, 68) |  |
|  | Discernment (68) |  |
|  | Time management skills (29, 62, 104) | - P-MEX (62, 104) - Time management inquiry form (104) |
|  | Cultural competence (104) | - Cultural competence self-assessment questionnaire (104) |
|  | Organisational skills (29, 68, 95, 99, 127, 128, 130) |  |
| Clinical competence | Risk assessment (29, 127) | - Peer-assessment (104) - OSCE (104) - Simulated patients rating scale (104) |
|  | Decision making (29, 68, 95, 99, 127, 128, 130)   - Clinical decision making (29) - Ethical decision making (68) |  |
|  | Situational awareness (29) |  |
|  | Quality and error management (29) |  |
|  | Procedural skills (29) |  |
|  | Diagnostic skills (104)   - Peer-assessment (104) |  |

##### Is

| Types of values | | | Tools of assessment |
| --- | --- | --- | --- |
| Personal values | Interpersonal values | Ethical values |  |
| - Excellence (29, 107, 135) - Altruism (29, 68, 99, 107, 109, 135) - Honor (135) - Confidence (29) - Innovative (29) - Empathy (5, 68, 94, 104) - Caring (99, 104) - Compassion (29, 99, 104) - Interest and curiosity (68) - Resilience (68, 69) - Adaptability (68) - Reflective (68) - Responsibility (29, 68, 94, 99, 140) - Trustworthy (29, 68, 99) - Capacity for improvement (68) - Openness (99) - Self-awareness (29, 68, 104, 109) - Honesty (68, 99, 104) - Integrity (29, 99, 104, 109, 135) - Politeness, courtesy, patience (104) - Accountability (29, 104, 107, 109, 135) - Humanism (29) | - Respect for others (29, 99, 104, 109, 135) - Teamwork (29, 99, 104, 135) | - Cultural sensitivity (29) - Equity (29) - Confidentiality (29) - Ethical and moral standards (99, 104) - Respect for privileges and code of conduct (104) - Honouring commitment (68, 99) - Competence (99) - Autonomy (99) - Respect for diversity and uniqueness (104) | - Moral reasoning assessments (73) - Written exams (73) - Reflections (5, 73) - Peer evaluations (68) - Moral judgement interview (114) - Defining issues test (63, 114, 120) - Interpersonal reactivity index (5) - Balanced emotional empathy scale (5) - Toronto empathy questionnaire (5) - Empathy quotient (5) - Social empathy index (5) - Empathic tendency scale (5) - Truax accurate empathy scale (5) - Empathy skill scale (5) - Empathy communication skill scale(5) |

| Types of attitudes | | | Tools used to assess attitudes |
| --- | --- | --- | --- |
| Attitudes towards other people | Attitudes towards oneself | Attitudes towards issues |  |
| - Interaction with patient (94) - Focus on patient’s needs above self (29) - Empathises and respects patient’s dignity (29) - Teamwork competencies (94, 141) - Service-oriented (68) - Emotional intelligence (68) - Cultural competence (68) - Humanism (29, 68, 107) | - Upholding moral fiduciary relationship and duty (29) - Upholding justice and social responsibility (29) - Commitment to lifelong learning (29) - Commitment to profession (29) - Self-motivation (29) - Motivation to Continuously improve (104, 135) | - Views towards unprofessional behaviors (29, 131) - Attitudes toward professionalism in medical education (121) - Attitudes towards empathy (114) | - Jefferson scale of Empathy (5, 94, 114) - Dundee Polyprofessionalism inventory (131) - PSCOM Professionalism questionnaire (121) - Attitudes-towards-professionalism scale (94) - Time Management Inquiry Form (104) - Pharmacy Professionalism Instrument (104) - Cross-cultural adaptability inventory (104) - Cultural competence self-assessment questionnaire (104) - Interpersonal Reactivity Index (104) - Penn State College of Medicine Professionalism Questionnaire (104) |
